# Identification of Hub Genes and Pathways Associated With Idiopathic Pulmonary Fibrosis *via* Bioinformatics Analysis

**DOI:** 10.3389/fmolb.2021.711239

**Published:** 2021-08-12

**Authors:** Hanxi Wan, Xinwei Huang, Peilin Cong, Mengfan He, Aiwen Chen, Tingmei Wu, Danqing Dai, Wanrong Li, Xiaofei Gao, Li Tian, Huazheng Liang, Lize Xiong

**Affiliations:** ^1^ Department of Anesthesiology and Perioperative Medicine, School of Medcine, Shanghai Fourth People’s Hospital, Tongji University, Shanghai, China; ^2^ Translational Research Institute of Brain and Brain-Like Intelligence, School of Medcine, Shanghai Fourth People’s Hospital, Tongji University, Shanghai, China; ^3^ Clinical Research Center for Anesthesiology and Perioperative Medicine, Tongji University, Shanghai, China

**Keywords:** idiopathic pulmonary fibrosiss, differentially expressed gene, hub genes, bioinformatics, WGCNA (weighted gene Co-expression network analyses), PPI

## Abstract

Idiopathic pulmonary fibrosis (IPF) is a progressive disease whose etiology remains unknown. The purpose of this study was to explore hub genes and pathways related to IPF development and prognosis. Multiple gene expression datasets were downloaded from the Gene Expression Omnibus database. Weighted correlation network analysis (WGCNA) was performed and differentially expressed genes (DEGs) identified to investigate Hub modules and genes correlated with IPF. Gene Ontology (GO), Kyoto Encyclopedia of Genes and Genomes (KEGG) enrichment analysis, and protein-protein interaction (PPI) network analysis were performed on selected key genes. In the PPI network and cytoHubba plugin, 11 hub genes were identified, including ASPN, CDH2, COL1A1, COL1A2, COL3A1, COL14A1, CTSK, MMP1, MMP7, POSTN, and SPP1. Correlation between hub genes was displayed and validated. Expression levels of hub genes were verified using quantitative real-time PCR (qRT-PCR). Dysregulated expression of these genes and their crosstalk might impact the development of IPF through modulating IPF-related biological processes and signaling pathways. Among these genes, expression levels of COL1A1, COL3A1, CTSK, MMP1, MMP7, POSTN, and SPP1 were positively correlated with IPF prognosis. The present study provides further insights into individualized treatment and prognosis for IPF.

## Introduction

Idiopathic pulmonary fibrosis (IPF), a chronic and progressive lung disease of unknown etiology (King et al., 2014; Martinez et al., 2017; Raghu et al., 2018), is characterized by diffuse alveolar inflammation and alveolar structural damage (Xu et al., 2016; Claude and Harold, 2018; David et al., 2019). The global annual incidence of IPF is 0.2–93.7 per 100,000 (Hutchinson et al., 2015) and the median survival time is only 2–3 years after diagnosis is confirmed (Tran and Suissa, 2018; Schäfer et al., 2020). Clinical biomarkers reliably reflecting the progression of IPF are small in number. In terms of treatment, medications recommended by current clinical guidelines - nintedanib and pirfenidone have a good therapeutic effect on IPF in general (Maher et al., 2019; Saito et al., 2019; Behr et al., 2021), but not that effective for late stage IPF. Therefore, it is important to illuminate the pathogenesis of IPF and to explore potential biomarkers for early stage IPF in order to improve the therapeutic effect on IPF.

In recent years, the amount of biological data for research has dramatically increased with the development of transcriptomic analysis, providing new viewpoints for exploring the etiology, pathogenesis, and novel targets for clinical treatment (DePianto et al., 2015; Wang et al., 2017). Previous studies primarily tested individual genes in diseased conditions. However, genes with similar expression patterns are likely to be tightly co-regulated *in vivo* with closely related functions, expressed in the same signaling pathways or processes (Pei et al., 2017). Identifying these genes and their interactions will provide further understanding of biological pathways related to IPF.

In the present study, multiple bioinformatic methods were used to search for hub genes significantly correlated with the occurrence and prognosis of IPF. Their expression has also been validated using quantitative real-time PCR (qRT-PCR). This work will provide further insights into the underlying molecular mechanisms of IPF and potential molecular targets for developing novel interventional strategies.

## Materials and Methods

### Acquisition of Microarray Data

In this study, seven gene array expression Series Matrix Files of IPF containing GSE110147, GSE53845, GSE24206, GSE32537, GSE68239, GSE10667, and GSE70866 were obtained from the Gene Expression Omnibus (GEO) repository (https://www.ncbi.nlm.nih.gov/geo/). The main features of these 7 datasets were shown in Table 1. These profiles consisted of gene expression matrices, probe sets, and clinical characteristics. Subsequent analyses were conducted on these datasets.

**TABLE 1:** The main features of 7 selected datasets included in this analysis.

GEO datasets	Platform	Sample size		Publication years	Regions
		IPF	Normal		
GSE110147	GPL6244	22	11	2018	London
GSE53845	GPL6480	40	8	2014	South San Francisco
GSE24206	GPL570	11	6	2011	Durham
GSE32537	GPL6244	119	50	2013	Aurora
GSE68239	GPL1708	10	10	2017	Giessen
GSE10667	GPL153	23	15	2003	Bern
GSE70866	GPL17077	112	20	2018	Hannover

### Removal of the Batch Effect

All of the raw data of GSE110147, GSE53845, GSE24206, GSE32537, and GSE68239 were merged and normalized into the training group using the RMA algorithm provided by the Limma package 3.40.6 of R, followed by removing the batch effect using R package SVA. The principal component analysis (PCA) was used to estimate whether the batch effect was removed. The validation group consisted of data from GSE10667 and GSE70866.

### Identification of Significant Modules Using the Weighted Correlation Network Analysis

The WGCNA package was used to determine key genes significantly associated with IPF in the training and validation groups. The best soft threshold power was set to identify the module-trait relationship, module membership (MM), and gene significance (GS). In brief, a weighted adjacency matrix was first constructed based on the selected soft threshold power. Subsequently, the connectivity per gene was deduced through calculating connection strengths with other genes. After validating the module structure preservation using the module preservation R function, the gene expression profile of each module was summarized by the module eigengene on whom IPF traits were regressed in the Limma R package (Huang et al., 2020). The IPF-related module was selected with the highest coefficient square (r^2^) and the *p* value <0.001.

### Identification of Differentially Expressed Genes

The Limma package 3.40.6 was used to compare DEGs between the normal group and the IPF group in the training group. The Benjamini-Hochberg’s method was used to control the false discovery rate (FDR), and DEGs were selected through adopting the commonly used threshold of FDR *p* value <0.01 and |Log2 fold-change| > 1. The volcano plot of DEGs was generated by the ffplot2 package, and a heatmap of DEGs was produced using the pheatmap package in the R software.

### Identification of Key Genes and Gene Set Enrichment Analysis

An online tool (http://bioinformatics.psb.ugent.be/webtools/Venn/) was employed to construct a Venn diagram to overlap the key genes in the significant modules and DEGs in the training group. The analyses of key genes of Gene Ontology (GO) and the Kyoto Encyclopedia of Genes and Genomes (KEGG) pathways were conducted using the GOplot, clusterProfiler, DOSE, colorspace, and the stringi packages in the R software.

### Protein-Protein Interaction Network Analysis

The Search Tool for the Retrieval of Interacting Genes (STRING, https://www. string-db. org/) was used to identify functional interactions between the products of the key genes in the training group. The PPI network was constructed using STRING by adopting the default threshold of a combined score >0.6. Then, the number of nodes of all the related proteins in the PPI network was counted using the R software and visualized using Cytoscape 3.8.2.

### The Correlation Between Hub Genes

The ggcorrpolt and the ggthemes of the R package were used to determine the correlation between hub genes in the training and the validation groups, respectively. *p* value <0.01 was considered statistically significant.

### Ethics Statement and Animal Treatment

In this study, 12 8 week-old male C57BL/6 mice weighing 20–25 g were obtained from Shanghai SLAC Laboratory Animal Ltd., China. Mice were bred at 22–24°C under a 12 h/12 h light/dark cycle and with free access to food and water. All procedures were implemented in conformity to the guidelines for animal care published by the United States’ National Institutes of Health (NIH) for animal care (Guide for the Care and Use of Laboratory Animals, Department of Health and Human Services, NIH publication No. 86–23, revised 1985). All procedures were approved by Renji hospital, Shanghai Jiao Tong University School of Medicine, Shanghai, China (approval number: RJ-20170930). 5 mg/kg LPS was intraperitoneally injected for 5 consecutive days to 6 mice to induce pulmonary fibrosis as reported in our previous article (Wan et al., 2019). The other 6 mice served as control. The lung tissue of all mice was collected 30 days after LPS injection.

### Hematoxylin and Eosin, Masson’s Trichrome Staining and Immunohistochemistry

Lung tissue collected from both control and LPS injected mice was fixed in 4% paraformaldehyde solution for overnight, followed by dehydration in 70% ethanol and embedding in the paraffin wax. 5 μm thick sections were cut and subject to H&E and Masson’s trichrome staining, respectively, as previously described (Wan et al., 2019). Expression of α-SMA in the lung tissue was evaluated using immunohistochemical staining. Sample sections were deparaffinized and incubated with 5% goat serum containing 0.1% Triton X-100 at room temperature for 2 h, followed by sequential incubation in the primary (rabbit anti-α-SMA, 1:2000, CST, Cat No 19245) and secondary antibody solutions (goat anti-rabbit IgG, 1:1000, Jackson Immunoresearch Laboratory, Cat No 111-035-003) antibodies, each for 2 h. After 3 rinses with 1x PBS, the sections were incubated in a 3,3,-diaminobenzidine (DAB) reaction complex (Vector lab, Burlingame, CA, United States) until an optimal colour developed. At the end of the procedure, the sections were mounted and dehydrated before being coverslipped.

### Quantitative Real-Time PCR and Statistical Analysis

Total RNA of the mouse lung tissue was isolated using Trizol (Thermofisher) according to the manufacturer’s instructions. Primer Script RT reagent kit (Takara, Japan) was used to synthesize complementary DNA, and real-time PCR was run on a Quant Studio 1 real-time PCR system (Thermofisher, United States) using the TB Green Premix Ex Taq^TM^ kit (Takara, Japan). Glyceraldehyde-3-phosphate dehydrogenase (GAPDH) is stably expressed in the lung tissue during the process of pulmonary fibrosis. Therefore, it serves as a reliable endogenous control in the reverse transcription-polymerase chain reaction (Loitsch et al., 1999; Cruz-Bermúdez et al., 2019). The 2^−△△Ct^ method was employed to assess relative expression levels of genes of interest. All of the data were presented as mean ± standard error of mean (SEM) and analyzed using the GraphPad PRISM8 software (United States). Two-tailed Student’s *t* test was used to compare between the two groups. *p* < 0.001 was considered statistically significant. Sequences of the primers could be found in Table 2.

**TABLE 2:** Real-time PCR primers.

Gene		Primer
GAPDH	F	AGG​TCG​GTG​TGA​ACG​GAT​TTG
GAPDH	R	TGT​AGA​CCA​TGT​AGT​TGA​GGT​CA
COL1A1	F	GCT​CCT​CTT​AGG​GGC​CAC​T
COL1A1	R	CCA​CGT​CTC​ACC​ATT​GGG​G
COL1A2	F	GTA​ACT​TCG​TGC​CTA​GCA​ACA
COL1A2	R	CCT​TTG​TCA​GAA​TAC​TGA​GCA​GC
COL3A1	F	CCT​GGC​TCA​AAT​GGC​TCA​C
COL3A1	R	CAG​GAC​TGC​CGT​TAT​TCC​CG
COL14A1	F	TTT​GGC​GGC​TGC​TTG​TTT​C
COL14A1	R	CGC​TTT​TGT​TGC​AGT​GTT​CTG
COL15A1	F	GCG​GAG​TCG​GGT​TTC​AGA​G
COL15A1	R	TAC​TTC​GCC​CGC​AGA​ACA​AA
MMP1	F	GGA​CAA​GCA​GTT​CCA​AAG​GC
MMP1	R	GAT​GCT​TAG​GGT​TGG​GGT​CT
MMP7	F	CTG​CCA​CTG​TCC​CAG​GAA​G
MMP7	R	GGG​AGA​GTT​TTC​CAG​TCA​TGG
CTSK	F	GAA​GAA​GAC​TCA​CCA​GAA​GCA​G
CTSK	R	TCC​AGG​TTA​TGG​GCA​GAG​ATT
CDH2	F	AGC​GCA​GTC​TTA​CCG​AAG​G
CDH2	R	TCG​CTG​CTT​TCA​TAC​TGA​ACT​TT
ASPN	F	AAG​GAG​TAT​GTG​ATG​CTA​CTG​CT
ASPN	R	ACA​TTG​GCA​CCC​AAA​TGG​ACA
POSTN	F	CCT​GCC​CTT​ATA​TGC​TCT​GCT
POSTN	R	AAA​CAT​GGT​CAA​TAG​GCA​TCA​CT
SPP1	F	AGC​AAG​AAA​CTC​TTC​CAA​GCA​A
SPP1	R	GTG​AGA​TTC​GTC​AGA​TTC​ATC​CG

### Survival Analysis of Hub Genes

The Kaplan-Meier method (K-M method; product-limit method) is suitable for analysis with a small sample size. Survival analysis was performed using survfit R package in the R software. The difference in survival curves for IPF patients with different expression levels of hub genes was analyzed using the log-rank method provided by the survival package.

## Results

### Removal of Batch Effects by Cross-Platform Normalization

**FIGURE 1:**
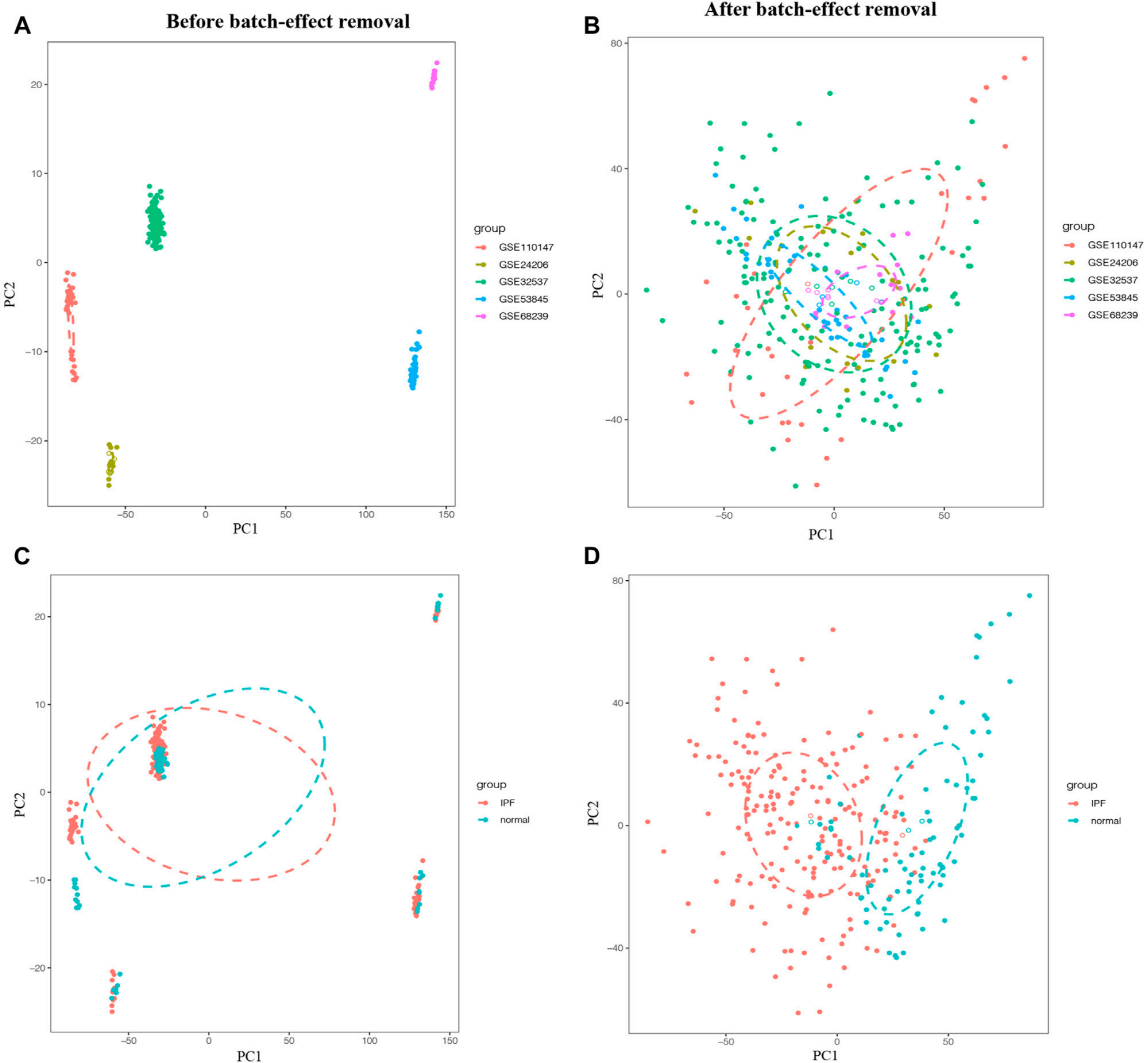
Principal component analysis (PCA) of the training group. The points of the scatter plots represented the samples without **(A,C)** and with **(B,D)** the removal of batch effect based on the top two principal components (PC1 and PC2) of gene expression profiles. Samples from five different datasets were color coded **(A,B)**, and the colors represented the IPF group and the normal group, respectively **(C,D)**.

To eliminate batch effects from different platforms and batches of datasets, the ComBat function of SVA package was used (Chen et al., 2020). Before the removal of batch effects, samples were clustered by batches based on the first two principal components (PC) of unnormalized expression values (Figures 1A,C). In contrast, the scatter plot of principal component analysis (PCA) based on normalized expression showed that batch processing effects from different platforms were eliminated (Figures 1B,D). These results confirmed that cross-platform standardization successfully eliminated batch effects.

### Identification of IPF-Associated Weighted Correlation 26 Network Analysis Modules

**FIGURE 2:**
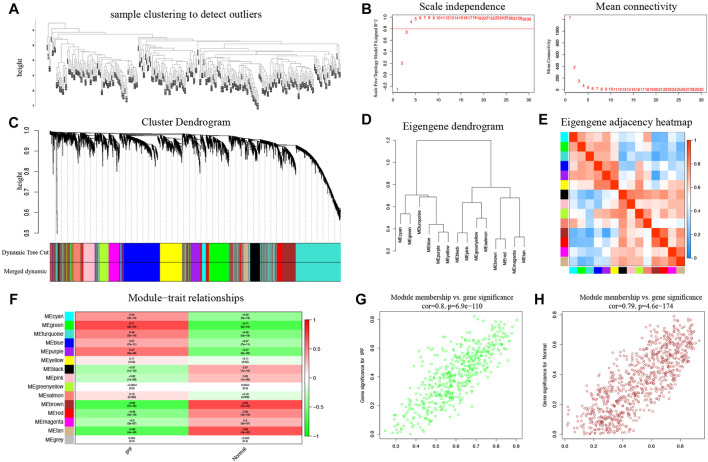
Key modules identified by WGCNA in the training group. **(A)** Sample clustering to detect outliers. **(B)** Determination of soft-thresholding power. **(C)** Clustering dendrogram. Both of the original and the merged modules were presented. **(D)** Dendrogram of consensus module eigengenes obtained from WGCNA on the consensus correlation. **(E)** Dendrogram of merged module eigengenes obtained from WGCNA. **(F)** The module-trait relationships were evaluated by correlating module eigengenes with clinical traits. **(G)** Scatter plots of module eigengenes in the green modules. **(H)** Scatter plots of module eigengenes in the brown modules.

A total of 287 samples and 6,263 genes retrieved from the training group were used for the co‐expression network analysis (Figure 2A). An eigengene correlation coefficient square (r^2^) of 0.8 and a soft threshold power of 3 were set to identify the module-trait relationship (Figure 2B). A hierarchical clustering tree was constructed following a dynamic hybrid cut (Figure 2C). The eigengene dendrogram and heatmap were used to quantify module similarity by eigengene correlation (Figures 2D,E). Among the 15 gene modules identified (Figure 2F), the green module (Figure 2G) showed a relatively higher positive correlation with the IPF group (cor = 0.71, *p* < 0.001), and the brown module (Figure 2H) exhibited significantly positive correlation with the IPF group (cor = −0.69, *p* < 0.001).

### Idiopathic Pulmonary Fibrosis-Associated Key Genes and Their Functions

**FIGURE 3:**
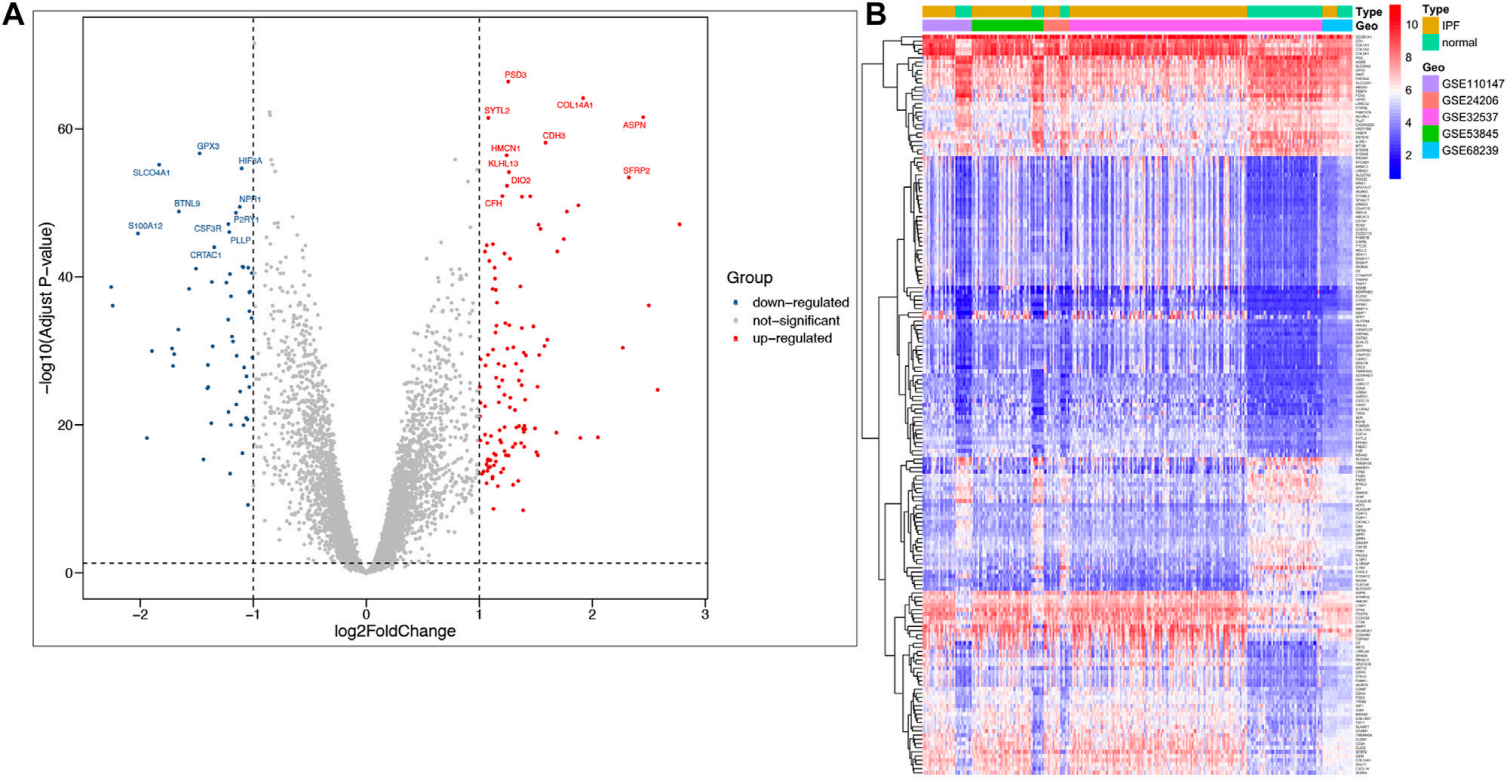
Identification of DEGs in IPF and the enrichment of these genes in the training group. **(A)** Volcano plot of all DEGs. **(B)** Heatmap of all DEGs.

**FIGURE 4:**
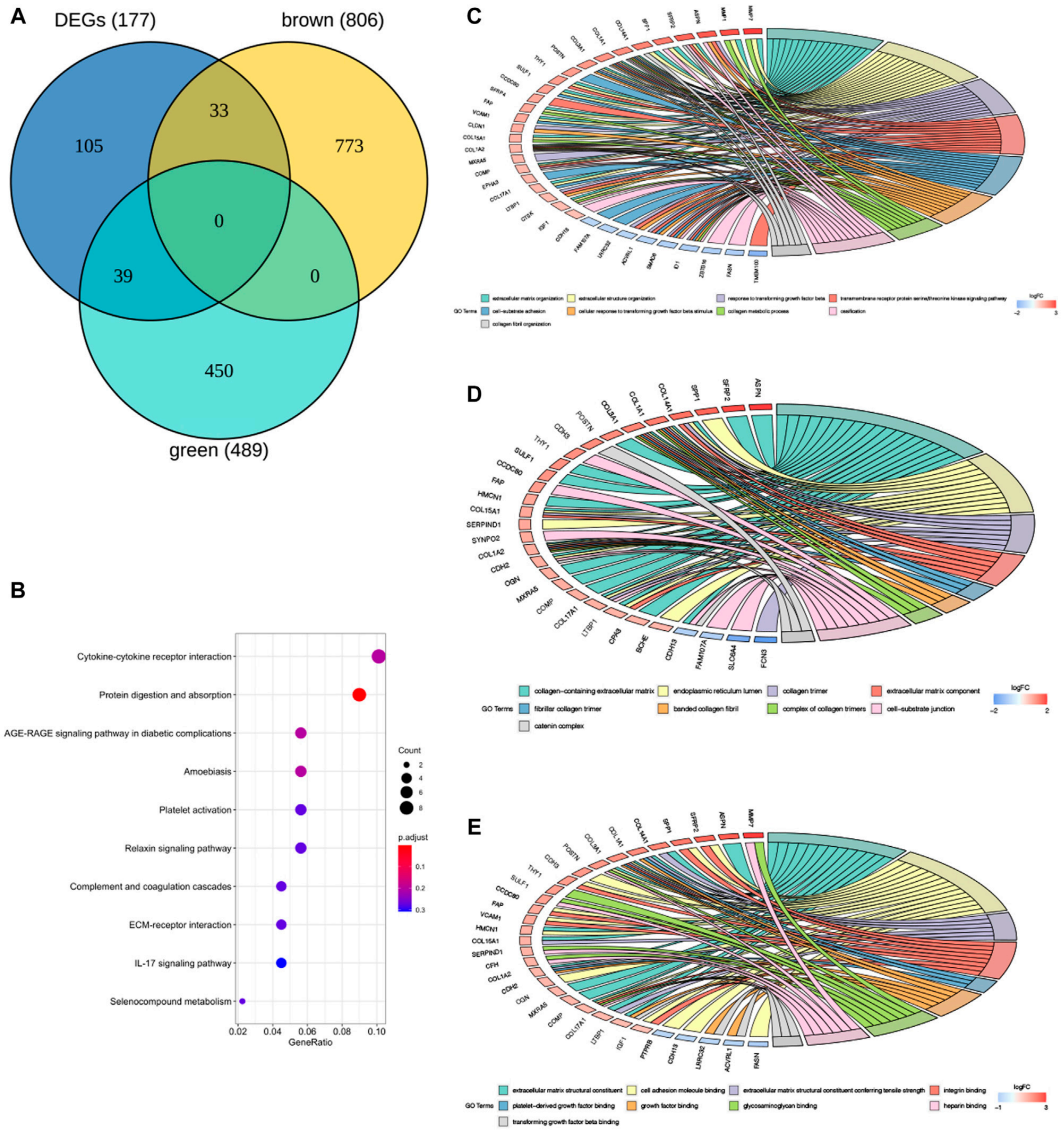
GO and KEGG enrichment analysis of key genes in the training group. **(A)** The Venn diagram demonstrating key genes in the training group. **(B)** Kyoto encyclopedia of genes and genomes (KEGG) analysis of key genes **(E)**. Gene ontology (GO) analysis of key genes in the training group **(C–E)**, **(C)** BP: biological process, **(D)** CC: cellular component, **(E)** MF: molecular function.

A total of 177 DEGs, including 121 up-regulated and 56 down-regulated genes, were identified in the training group (Figures 3A,B, Table 3). Among them, 72 genes were determined as key genes because they were shared by the green and brown modules (Figure 4A and Table 4). KEGG analysis revealed that the most notably enriched pathways were protein digestion and absorption and focal adhesion (Figure 4B, Supplementary Table S1). GO analysis showed that these genes were mainly involved in nine cellular components (Figure 4D), nine molecular functions (Figure 4E), and nine biological processes, including extracellular matrix organization (CCDC80, COL14A1, COL15A1, COL17A1, COL1A1, COL1A2, COL3A1, COMP, CTSK, FAP, MMP1, MMP7, POSTN, SFRP2, SPP1, SULF1, and VCAM1), extracellular structure organization (CCDC80, COL14A1, COL15A1, COL17A1, COL1A1, COL1A2, COL3A1, COMP, CTSK, FAP, MMP1, MMP7, POSTN, SFRP2, SPP1, SULF1, and VCAM1), response to TGF beta (ACVRL1, ASPN, CLDN1, COL1A1, COL1A2, COL3A1, ID1, LRRC32, LTBP1, MXRA5, POSTN, SMAD6), and others (Figure 4C), indicating that these genes could play important roles in the occurrence of IPF through influencing these biological processes and molecular functions.

**TABLE 3:** DEGs in the training group.

DEGs	logFC	T	adj.*P*.Val	DEGs	logFC	T	adj.*P*.Val
MMP7	2.77203	17.63598	1.45E-45	TTC25	1.13038	8.34492	1.63E-14
MMP1	2.57792	11.49570	2.86E-24	SERPINB3	1.12534	6.17062	6.96E-09
CP	2.50036	14.67839	3.84E-35	COMP	1.12175	13.03885	1.84E-29
ASPN	2.45043	21.58471	2.65E-59	EPHA3	1.12035	16.91606	5.04E-43
SFRP2	2.32371	19.35112	1.18E-51	COL17A1	1.11795	15.28514	2.69E-37
KRT5	2.27018	13.10825	1.06E-29	CAPSL	1.11736	7.81414	4.85E-13
SPP1	2.04957	9.57238	3.87E-18	TEKT1	1.11608	7.72356	8.51E-13
COL14A1	1.91875	22.30443	1.38E-61	SLN	1.10315	9.63114	2.53E-18
PROM1	1.89389	9.54929	4.54E-18	ARMC4	1.09898	8.57409	3.58E-15
CD24	1.87693	18.33103	5.22E-48	C9orf135	1.09611	8.27343	2.58E-14
COL1A1	1.77486	18.10569	3.19E-47	CNTN3	1.08825	16.30609	7.36E-41
IL13RA2	1.74783	17.10082	1.11E-43	C10orf107	1.08106	8.24539	3.09E-14
COL3A1	1.69034	16.64818	4.49E-42	SYTL2	1.07978	21.56171	2.75E-59
SPAG6	1.68222	9.76954	9.58E-19	CCDC113	1.07764	8.61839	2.67E-15
POSTN	1.60165	13.40728	9.90E-31	FGF14	1.07645	12.83779	8.78E-29
CDH3	1.58629	20.63370	5.59E-56	DNAH11	1.07410	8.54680	4.30E-15
TDO2	1.57672	13.17209	6.44E-30	C6orf118	1.07219	8.08930	8.48E-14
THY1	1.54091	17.47384	5.18E-45	STK33	1.07050	9.32753	2.14E-17
SPATA18	1.53005	12.83180	9.10E-29	LTBP1	1.06576	16.87593	6.95E-43
SULF1	1.52483	17.62247	1.58E-45	SCGB3A1	1.06404	7.50102	3.34E-12
EFCAB1	1.51802	8.79445	8.24E-16	CLCA2	1.06387	8.38614	1.24E-14
WDR49	1.51485	11.61863	1.12E-24	CTSK	1.05448	12.43521	2.01E-27
ARMC3	1.50479	8.93218	3.24E-16	GPR87	1.05106	9.68485	1.74E-18
LRRIQ1	1.49233	9.94574	2.73E-19	CPA3	1.05092	10.85025	3.72E-22
CCDC80	1.47922	13.90511	1.87E-32	TRIM2	1.05023	16.64325	4.60E-42
FNDC1	1.45118	18.65814	3.50E-49	MDH1B	1.03820	8.05977	1.03E-13
MMP13	1.41253	12.81749	1.01E-28	RP1	1.02349	7.95282	2.03E-13
DSC3	1.40548	9.91334	3.42E-19	BCHE	1.01119	12.67214	3.19E-28
TMPRSS4	1.40534	11.10442	5.56E-23	FAM83D	1.01104	10.98620	1.34E-22
KRT15	1.39987	10.04997	1.28E-19	IGF1	1.00989	9.43908	9.76E-18
TSPAN1	1.39759	9.16860	6.38E-17	SERPINI2	1.00692	10.43532	7.83E-21
VSIG1	1.39411	9.79879	7.76E-19	NEK11	1.00509	11.60561	1.23E-24
SFRP4	1.39356	12.92391	4.51E-29	PTPRB	−1.00886	−12.73755	1.89E-28
SLC27A2	1.38762	9.93672	2.91E-19	GPR4	−1.01324	−15.86403	2.58E-39
MSMB	1.38711	6.09267	1.06E-08	CDH13	−1.01489	−14.21637	1.55E-33
LRRC17	1.37754	18.64756	3.67E-49	FAM107A	−1.02720	−15.18956	5.82E-37
FAP	1.37635	13.84266	3.06E-32	LRRC32	−1.03409	−14.46684	2.04E-34
VCAM1	1.37488	11.67872	7.09E-25	AFF3	−1.03498	−15.16171	7.31E-37
TMEM45A	1.37431	12.22798	1.00E-26	FPR1	−1.03536	−11.60303	1.25E-24
SPAG17	1.36994	9.32741	2.14E-17	PAPSS2	−1.04447	−16.05832	5.45E-40
HSPA4L	1.36353	15.37477	1.31E-37	ANKRD1	−1.04684	−6.38902	2.14E-09
CYP24A1	1.35190	10.04109	1.37E-19	PROK2	−1.05142	−10.31854	1.83E-20
SLITRK6	1.35026	11.86088	1.75E-25	PLA2G4F	−1.05968	−12.02025	5.07E-26
C20orf85	1.34465	7.61371	1.68E-12	HSD17B6	−1.06280	−10.37877	1.17E-20
WDR78	1.32791	9.98149	2.11E-19	CA4	−1.08075	−12.36029	3.54E-27
SLAMF7	1.32516	12.50422	1.19E-27	CACNA2D2	−1.08576	−10.07912	1.04E-19
GSTA1	1.31783	9.29345	2.71E-17	GRASP	−1.08796	−16.07649	4.82E-40
FANK1	1.31526	10.69559	1.15E-21	ACVRL1	−1.09478	−16.10034	4.06E-40
CLDN1	1.30119	13.12259	9.55E-30	FKBP5	−1.09614	−8.88744	4.39E-16
SCGB1A1	1.29900	7.41937	5.46E-12	HIF3A	−1.10235	−19.68359	8.92E-53
FAM81B	1.29639	9.15948	6.76E-17	SLC39A8	−1.11561	−11.43034	4.67E-24
RGS22	1.27438	11.18547	3.05E-23	NPR1	−1.11946	−18.27731	7.94E-48
LRRN1	1.27217	16.38499	3.81E-41	SMAD6	−1.14680	−12.80512	1.11E-28
MNS1	1.26945	10.80550	5.15E-22	CHI3L2	−1.14961	−10.91890	2.23E-22
CXCL14	1.26547	13.94959	1.33E-32	P2RY1	−1.15272	−18.05941	4.57E-47
KLHL13	1.26199	19.54838	2.33E-52	IL18R1	−1.17874	−13.34223	1.66E-30
C6	1.25902	8.78218	8.94E-16	SLCO2A1	−1.18823	−13.52812	3.82E-31
PSD3	1.25559	22.93054	1.21E-63	ID1	−1.19708	−15.01403	2.47E-36
PRSS12	1.24824	13.07355	1.39E-29	ZBTB16	−1.19762	−10.08938	9.64E-20
DIO2	1.24480	19.04587	1.42E-50	PGC	−1.20442	−7.95816	1.96E-13
HMCN1	1.24180	20.16614	2.28E-54	FASN	−1.20615	−15.82625	3.34E-39
CHST9	1.23465	8.79836	8.04E-16	PLLP	−1.21218	−17.35871	1.35E-44
DZIP3	1.23050	14.02285	7.40E-33	HHIP	−1.21951	−10.61885	2.03E-21
SPATA17	1.22401	9.99867	1.86E-19	CSF3R	−1.22052	−17.64669	1.36E-45
CXCL13	1.22326	8.97533	2.42E-16	INMT	−1.22178	−14.15692	2.48E-33
NELL2	1.22284	11.29983	1.27E-23	S100A9	−1.23775	−15.51469	4.21E-38
COL15A1	1.22278	16.57565	8.04E-42	CRTAC1	−1.34543	−16.80469	1.23E-42
SERPIND1	1.22108	12.41441	2.34E-27	IL18RAP	−1.35876	−13.16085	7.03E-30
DNAH9	1.21265	8.18456	4.61E-14	FMO5	−1.36614	−15.53888	3.46E-38
CFH	1.20440	18.66791	3.37E-49	AGER	−1.37110	−10.15923	5.84E-20
HHLA2	1.20322	11.86874	1.65E-25	ABCA3	−1.39859	−11.60612	1.22E-24
SOX2	1.19383	9.35110	1.82E-17	CLEC4E	−1.40101	−12.45507	1.74E-27
STOML3	1.19238	8.01573	1.36E-13	IL1RL1	−1.40643	−11.55651	1.78E-24
WDR63	1.18491	9.45155	8.98E-18	CPB2	−1.44059	−8.61043	2.81E-15
DNAH7	1.18030	9.86856	4.69E-19	GPX3	−1.47462	−20.23649	1.40E-54
CLIC6	1.17339	11.61737	1.13E-24	S100A8	−1.50688	−16.02132	7.36E-40
AMPD1	1.17332	11.00342	1.17E-22	MGAM	−1.56789	−15.28718	2.67E-37
SYNPO2	1.16962	12.49577	1.27E-27	BTNL9	−1.65868	−18.10805	3.19E-47
LRRC46	1.16388	7.35442	8.10E-12	PEBP4	−1.66297	−13.78816	4.80E-32
COL1A2	1.15626	14.78199	1.68E-35	PLA2G1B	−1.69918	−12.86725	7.02E-29
GEM	1.15200	13.93094	1.53E-32	MT1M	−1.70969	−12.41717	2.30E-27
MS4A2	1.14954	11.96580	7.75E-26	VIPR1	−1.71810	−13.07951	1.33E-29
CDH2	1.14648	15.24888	3.59E-37	SLCO4A1	−1.83259	−19.81841	3.06E-53
NEK10	1.14598	8.80644	7.61E-16	TMEM100	−1.89595	−12.98297	2.82E-29
ABCA13	1.14585	8.51578	5.27E-15	SLC6A4	−1.94099	−9.54127	4.80E-18
OGN	1.14330	13.67341	1.20E-31	S100A12	−2.02037	−17.30450	2.09E-44
CRISPLD1	1.13884	15.66068	1.29E-38	FCN3	−2.24401	−14.66999	4.09E-35
MXRA5	1.13572	16.05886	5.45E-40	IL1R2	−2.25735	−15.35214	1.57E-37
CASC1	1.13124	8.86592	5.08E-16				

**TABLE 4:** Key genes in the training group.

Key genes	logFC	adj.*P*.Val	module	*p*.GS.IPF	*p*.MMgreen	*p*.MMbrown
MMP7	2.77202754	1.45E^-^45	brown	5.30E^-^47	2.48E^-^21	2.87E^-^73
MMP1	2.57792316	2.86E^-^24	brown	5.09E^-^25	3.50E^-^15	7.98E^-^75
ASPN	2.45042861	2.65E^-^59	green	2.79E^-^61	6.46E^-^72	1.87E^-^19
SFRP2	2.32371277	1.18E^-^51	green	2.87E^-^53	7.07E^-^69	6.49E^-^40
SPP1	2.04957433	3.87E^-^18	brown	1.00E^-^18	1.07E^-^11	2.99E^-^69
COL14A1	1.91875456	1.38E^-^61	green	6.58E^-^64	1.08E^-^106	7.07E^-^38
COL1A1	1.77486012	3.19E^-^47	green	8.58E^-^49	1.23E^-^59	9.40E^-^31
IL13RA2	1.74782674	1.11E^-^43	green	4.06E^-^45	9.74E^-^37	4.43E^-^43
COL3A1	1.6903409	4.49E^-^42	green	1.82E^-^43	5.66E^-^63	2.63E^-^26
POSTN	1.6016506	9.90E^-^31	green	1.02E^-^31	2.69E^-^63	9.45E^-^26
CDH3	1.58629259	5.59E^-^56	brown	5.03E^-^58	1.08E^-^27	1.24E^-^83
THY1	1.54091349	5.18E^-^45	green	1.61E^-^46	2.66E^-^67	3.47E^-^27
SULF1	1.52483345	1.58E^-^45	green	4.55E^-^47	2.16E^-^71	3.70E^-^47
CCDC80	1.47922228	1.87E^-^32	green	1.65E^-^33	3.35E^-^65	4.08E^-^28
FNDC1	1.45118385	3.50E^-^49	green	6.98E^-^51	3.32E^-^61	1.85E^-^19
SFRP4	1.39356095	4.51E^-^29	green	5.09E^-^30	5.13E^-^56	2.73E^-^15
LRRC17	1.37753844	3.67E^-^49	green	7.23E^-^51	5.29E^-^76	2.11E^-^24
FAP	1.37635339	3.06E^-^32	green	2.68E^-^33	1.79E^-^44	2.49E^-^22
VCAM1	1.37488333	7.09E^-^25	green	1.11E^-^25	4.12E^-^37	3.64E^-^20
CLDN1	1.30119139	9.55E^-^30	brown	9.80E^-^31	2.17E^-^17	1.81E^-^60
CXCL14	1.26547198	1.33E^-^32	brown	1.06E^-^33	3.12E^-^30	2.76E^-^48
PSD3	1.25559334	1.21E^-^63	green	2.08E^-^66	3.62E^-^45	7.85E^-^42
DIO2	1.24479786	1.42E^-^50	green	2.21E^-^52	1.58E^-^63	2.59E^-^43
HMCN1	1.24179754	2.28E^-^54	green	1.77E^-^56	2.66E^-^69	5.68E^-^19
COL15A1	1.22278349	8.04E^-^42	green	2.65E^-^43	1.17E^-^78	1.18E^-^33
SERPIND1	1.22107917	2.34E^-^27	green	3.01E^-^28	1.52E^-^17	2.45E^-^24
CFH	1.20439698	3.37E^-^49	green	5.13E^-^51	9.50E^-^71	6.31E^-^29
SYNPO2	1.16962063	1.27E^-^27	green	1.53E^-^28	6.24E^-^68	4.75E^-^17
COL1A2	1.15626233	1.68E^-^35	green	9.57E^-^37	3.82E^-^65	7.11E^-^18
GEM	1.15200269	1.53E^-^32	green	1.17E^-^33	5.45E^-^56	2.55E^-^18
MS4A2	1.14954201	7.75E^-^26	green	1.07E^-^26	2.78E^-^44	2.17E^-^11
CDH2	1.14647827	3.59E^-^37	green	1.87E^-^38	2.24E^-^52	2.85E^-^27
OGN	1.14329874	1.20E^-^31	green	9.85E^-^33	2.86E^-^70	5.44E^-^14
MXRA5	1.13572189	5.45E^-^40	green	1.96E^-^41	2.90E^-^56	6.45E^-^27
COMP	1.12174857	1.84E^-^29	green	1.81E^-^30	2.67E^-^32	1.30E^-^24
EPHA3	1.12035363	5.04E^-^43	green	1.33E^-^44	1.15E^-^75	2.16E^-^18
COL17A1	1.11795013	2.69E^-^37	brown	1.35E^-^38	1.07E^-^22	3.90E^-^66
SLN	1.10315195	2.53E^-^18	green	6.10E^-^19	2.72E^-^34	1.93E^-^26
SYTL2	1.07977843	2.75E^-^59	green	1.13E^-^61	2.54E^-^64	3.95E^-^48
LTBP1	1.06576443	6.95E^-^43	green	1.76E^-^44	5.44E^-^93	4.58E^-^23
CTSK	1.05448468	2.01E^-^27	green	2.38E^-^28	4.70E^-^37	2.13E^-^33
CPA3	1.05091777	3.72E^-^22	green	6.51E^-^23	1.78E^-^38	1.02E^-^10
BCHE	1.01119209	3.19E^-^28	green	3.40E^-^29	1.02E^-^50	2.49E^-^11
FAM83D	1.01103826	1.34E^-^22	green	2.25E^-^23	5.23E^-^32	6.98E^-^24
IGF1	1.00988527	9.76E^-^18	green	2.41E^-^18	2.62E^-^51	1.22E^-^25
PTPRB	−1.0088593	1.89E^-^28	brown	1.99E^-^29	6.74E^-^12	1.22E^-^93
CDH13	−1.0148952	1.55E^-^33	brown	9.83E^-^35	9.31E^-^09	1.60E^-^64
FAM107A	−1.0272098	5.82E^-^37	brown	2.77E^-^38	1.90E^-^20	1.28E^-^60
LRRC32	−1.0340983	2.04E^-^34	brown	1.23E^-^35	1.19E^-^18	1.13E^-^45
AFF3	−1.0349871	7.31E^-^37	brown	3.54E^-^38	3.94E^-^25	2.76E^-^71
PAPSS2	−1.0444732	5.45E^-^40	brown	1.79E^-^41	5.89E^-^24	3.39E^-^92
CA4	−1.0807466	3.54E^-^27	brown	4.41E^-^28	6.60E^-^26	9.75E^-^78
GRASP	−1.0879563	4.82E^-^40	brown	1.61E^-^41	1.50E^-^23	6.02E^-^55
ACVRL1	−1.094783	4.06E^-^40	brown	1.33E^-^41	3.35E^-^23	1.21E^-^102
HIF3A	−1.1023551	8.92E^-^53	brown	8.22E^-^55	1.77E^-^27	4.71E^-^84
NPR1	−1.1194655	7.94E^-^48	brown	1.26E^-^49	4.32E^-^21	6.18E^-^96
SMAD6	−1.1468095	1.11E^-^28	brown	1.23E^-^29	6.60E^-^23	6.82E^-^85
P2RY1	−1.1527251	4.57E^-^47	brown	8.37E^-^49	2.79E^-^29	1.28E^-^64
SLCO2A1	−1.1882355	3.82E^-^31	brown	3.35E^-^32	7.44E^-^15	9.97E^-^90
ID1	−1.197083	2.47E^-^36	brown	1.40E^-^37	1.37E^-^30	4.03E^-^49
ZBTB16	−1.1976268	9.64E^-^20	brown	2.13E^-^20	8.96E^-^15	7.42E^-^30
FASN	−1.2061532	3.34E^-^39	brown	1.49E^-^40	6.58E^-^34	9.60E^-^35
PLLP	−1.2121833	1.35E^-^44	brown	3.42E^-^46	1.56E^-^36	2.59E^-^89
INMT	−1.2217822	2.48E^-^33	brown	1.85E^-^34	1.02E^-^13	1.67E^-^83
CRTAC1	−1.3454348	1.23E^-^42	brown	4.15E^-^44	4.62E^-^50	1.30E^-^54
FMO5	−1.3661473	3.46E^-^38	green	1.85E^-^39	3.11E^-^33	1.02E^-^42
GPX3	−1.4746242	1.40E^-^54	brown	1.26E^-^56	4.55E^-^26	1.03E^-^90
BTNL9	−1.6586875	3.19E^-^47	brown	8.05E^-^49	2.28E^-^29	1.37E^-^91
VIPR1	−1.7181029	1.33E^-^29	brown	1.52E^-^30	8.84E^-^25	8.16E^-^109
TMEM100	−1.8959563	2.82E^-^29	brown	3.41E^-^30	5.53E^-^22	8.15E^-^97
SLC6A4	−1.9409992	4.80E^-^18	brown	1.25E^-^18	3.81E^-^17	1.51E^-^82
FCN3	−2.2440192	4.09E^-^35	brown	3.23E^-^36	2.01E^-^31	1.07E^-^76

### Identification of Hub Genes

**FIGURE 5:**
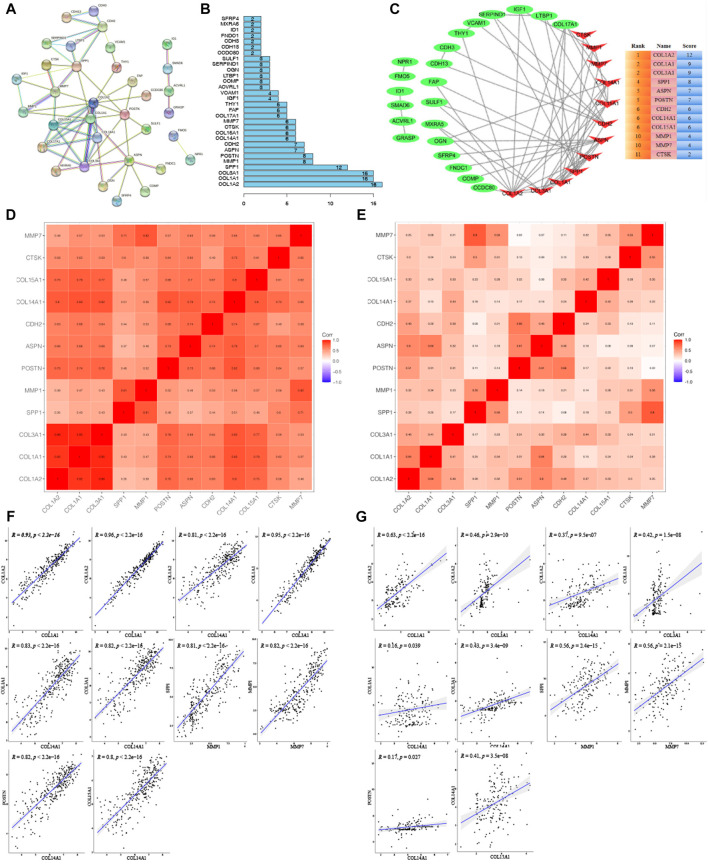
Cluster analysis of the PPI network in the training group. **(A)** Protein-protein interaction (PPI) network for key genes. **(B)** Histogram of key genes. **(C)** The hub gene network analyzed by Cytosacpe. **(D)** Correlation between hub genes in the training group. **(E)** Correlation between hub genes in the validation group. **(F)** Display of correlations between ten groups of genes with a correlation coefficient >0.8 in the training group. **(G)** Display of correlations between ten groups of genes in the validation group.

To explore hub genes, the PPI network analysis and cytoHubba plugin within cytoscape version 3.8.2 were performed based on the rank of the connection degree (number) of each gene. As shown in Figures 5A,B **,** nodes of 72 genes in the PPI network were identified according to the medium confidence ≥0.6. Finally, twelve hub genes were confirmed based on their node degrees within cytoHubba plugin (Figure 5A **)**, including collagen alpha-1 chain (I) (COL1A1), collagen alpha-2 chain (I) (COL1A2), collagen alpha-1 (III) chain (COL3A1), collagen alpha-1 chain (XIV) (COL14A1), collagen alpha-1 chain (XV) (COL15A1), periostin (POSTN), secreted phosphoprotein 1 (SPP1), matrix metallopeptidase 1 (MMP1), matrix metallopeptidase 7 (MMP7), asporin (ASPN), cadherin 2 (CDH2), and cathepsin K (CTSK). In addition, correlation analysis showed a highly consistent positive correlation between 12 hub genes in both the training group (Figures 5D,F) and the validation group (Figures 5E,G), such as COL1A2 and COL1A1, COL1A2 and COL3A1, COL1A2 and COL14A1, COL1A1 and COL3A1, COL1A1 and COL14A1, COL3A1 and COL14A1, SPP1 and MMP1, MMP1 and MMP7, POSTN and COL14A1, COL14A1 and COL15A1 (0.17 < cor <0.96, 2.2E-16 < *p* < 0.039), which further validated the results by PPI and cytoHubba plugin analysis (Figure 5C).

To further confirm the role of these 12 hub genes in IPF, another WGCNA for 6,252 genes obtained from GSE10667 of the training group was performed. It was found that the black and greenyellow modules, containing 12 hub genes, out of the 14 gene modules identified (**Figure S1a**) showed strong association with IPF (black module: cor = -0.63, *p* < 0.001; greenyellow module: cor = 0.81, *p* < 0.001) (Supplementary Figures S1B–D). These hub genes were involved in many important IPF-related biological processes, such as extracellular matrix organization, response to TGF beta, collagen metabolic process, fibrillar collagen trimer, etc (Supplementary Table S2 **)**. These results suggested that the abovementioned hub genes play important roles in the occurrence of IPF.

### Expression Validation of Hub Genes by Quantitative Real-Time -PCR

**FIGURE 6:**
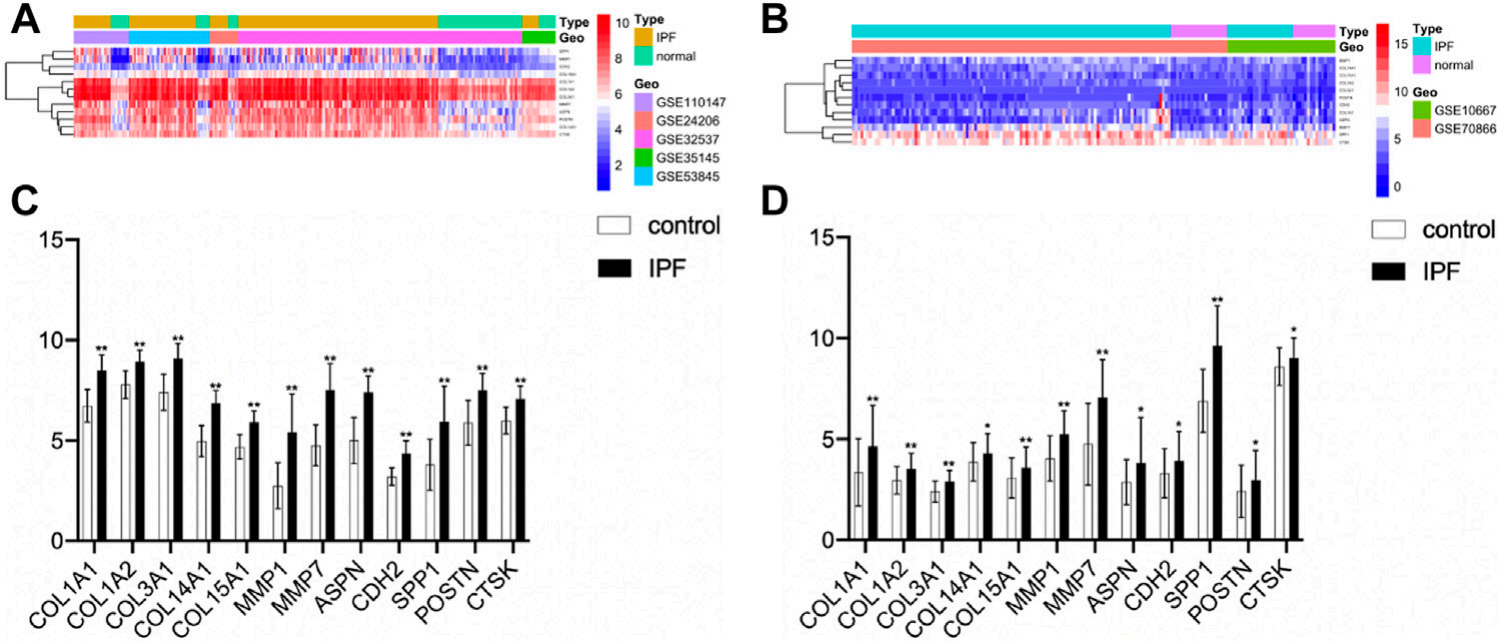
Correlation analysis of hub genes. **(A)** Heatmap of the training group. **(B)** Heatmap of the validation group. **(C)** Expression levels of twelve hub genes (healthy control samples vs IPF samples) from the training group. **(D)** Expression levels of twelve hub genes from the validation group (*p < 0.05, **p < 0.01).

**FIGURE 7:**
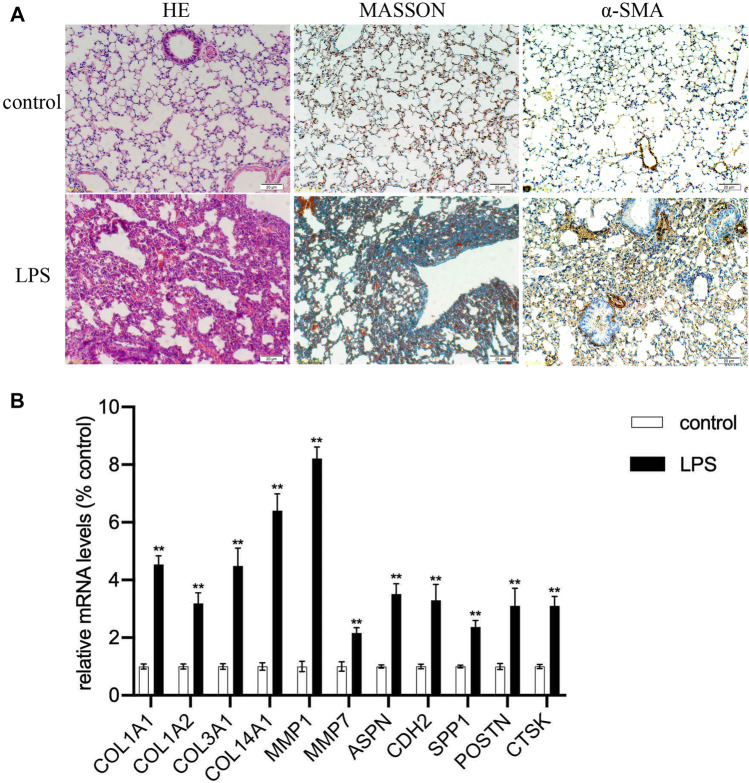
Expression validation of hub genes. **(A)** The severity of pulmonary fibrosis was determined by hematoxylin-eosin (HE) staining, collagen deposition was assessed by Masson’s trichrome staining, and α-SMA expression in the lung tissue was detected by immunohistochemistry (magnification, ×200). **(B)** Expression levels of COL1A1, COL1A2, COL3A1, COL14A1, MMP1, MMP7, ASPN, CDH2, SPP1, POSRN, CTSK mRNAs in lung tissue samples (**p < 0.01).

The clustering heatmap showed expression levels of hub genes in the training (Figure 6A) and the validation groups (Figure 6B). Expression levels of 12 hub genes showed consistent upregulation in both the training (Figure 6C) and the validation groups (Figure 6D). To further validate the results, qRT-PCR was conducted in the LPS-induced pulmonary fibrosis model. The modeling process was described in detail in our previous study (Wan et al., 2019). Firstly, mice were intraperitoneally injected with saline or LPS (5 mg/kg) for 5 consecutive days, and samples were collected on day 30 after LPS injection. H&E E/MASSON staining and α-SMA immunohistochemistry (Figure 7A) showed that the model of pulmonary fibrosis was successfully constructed. The lung tissue of 6 control and 6 LPS injected mice was collected. As shown in Figure 7B, expression levels of COL1A1, COL1A2, COL3A1, COL14A1, MMP1, MMP7, ASPN, CDH2, SPP1, POSTN, and CTSK were significantly increased in the lung tissue of LPS injected mice compared with that of control mice (*p* < 0.001), whereas the expression level of COL15A1 was undetectable in the lung tissue of either the control or LPS injected mice. These results demonstrated important roles COL1A1, COL1A2, COL3A1, COL14A1, MMP1, MMP7, ASPN, CDH2, SPP1, POSTN and CTSK play in IPF occurrence.

### Survival Analysis of Hub Genes in the Validation Group

**FIGURE 8:**
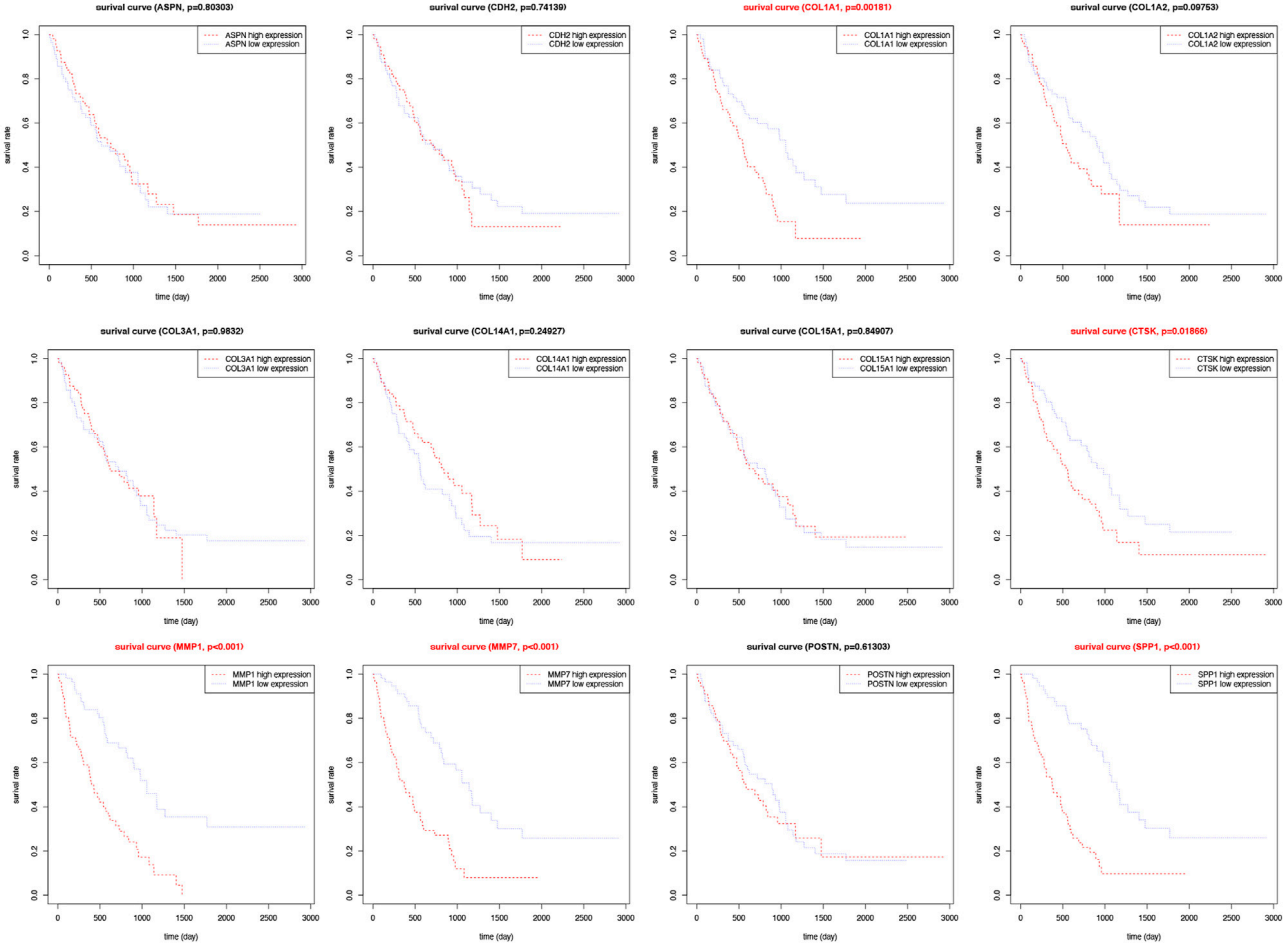
Survival analysis for hub genes in the validation group. Kaplan-Meier plot depicting association of the expression level of twelve individual hub genes with patient survival.

To explore the prognostic value of these 12 hub genes for IPF patients, overall survival analysis was performed using the GSE70866 dataset. It was found that IPF patients with high expression levels of COL1A1, CTSK, MMP1, MMP7, and SPP1 had poorer overall survival compared to those with low expression levels (*p* < 0.05). No significant correlation was found between expression levels of other hub genes and survival of IPF patients (*p* > 0.05, Figure 8).

## Discussion

Idiopathic pulmonary fibrosis (IPF) is an end-stage lung disease with a short survival time after diagnosis is confirmed (Liu et al., 2018). Therefore, it is vital to illuminate the underlying mechanism of IPF pathogenesis and to explore potential biomarkers for early diagnosis in order to improve the prognosis of IPF patients.

The present study used bioinformatic methods to screen IPF-related genes and tentatively tested their roles in the occurrence and prognosis of IPF based on the gene expression data of IPF patients in the GEO databases. Compared to other bioinformatics analyses, WGCNA is more valuable due to the comprehensive examination of links between co-expression modules and clinical traits with high reliability and biological significance (Wei et al., 2014; Kong et al., 2020; Lund et al., 2020). In the present study, 11 hub genes (COL1A2, COL1A1, COL3A1, SPP1, MMP1, POSTN, ASPN, CDH2, COL14A1, CTSK, MMP7) were found to be associated with the occurrence of IPF using WGCNA, differential gene expression analysis in the training datasets, PPI network analysis, and cytoHubba plugin analyses. Expression of these genes was validated in the GSE10667 dataset and in pulmonary fibrosis model mice using qRT-PCR. These results are consistent with those of previous studies in that COL1A1, COL1A2, COL3A1, COL14A1, POSTN, SPP1, MMP1, ASPN, MMP7, CDH2, and CTSK were up-regulated in a variety of samples from IPF patients and IPF-related mouse models, including blood, bronchoalveolar lavage, airway fibroblasts, and the lung tissue of human and mice, as well as involved in the occurrence of IPF (Supplementary Table S3) (Tzortzaki et al., 2003; Brule et al., 2005; Obermajer et al., 2006; Ivan et al., 2008; Naik et al., 2012; Vittal et al., 2013; Zhang et al., 2013; Hamai et al., 2016; Ghavami et al., 2018; Liu et al., 2018; Morimoto et al., 2018; Gao et al., 2019; Yu et al., 2020).

The collagen binding protein ASPN, a member of the family of leucine-rich repeat proteins, is produced by fibroblasts (Nakajima et al., 2007). A previous study showed that the expression of ASPN was up-regulated in the IPF(+) soluble fractions (Åhrman et al., 2018). COL1A1, a functional gene that encodes the alpha 1 chain of type I collagen, is the main constituent of ECM component (Li et al., 2020) and participates in cell proliferation, infiltration, metastasis, and angiogenesis. Its expression is related to many types of tumors (Bi et al., 2017; Ma et al., 2019; Dong et al., 2020). It is known that type III collagen interacts with type I and type II collagen in fibril formation and is an essential regulator of fibril diameter. The increase in type I, III, XIV, XV collagen content will lead to the formation of fibrils. Like many fibrotic disorders, IPF is characterized by enhanced deposition and remodeling of ECM (Tjin et al., 2017). Matrix metalloproteinases (MMPs) are a family of calcium- and zinc-dependent endopeptidases (Visse and Nagase, 2003) whose function is to regulate abnormal epithelial response to injury, fibroblast proliferation, extracellular matrix accumulation, and aberrant tissue remodeling (Ivan et al., 2008). MMPs play a role in different pathological processes such as atherosclerosis (Wågsäter et al., 2011), arthritis (Huang et al., 2017), tumor invasion, and pulmonary fibrosis (Ivan et al., 2008). POSTN plays a notable role in ECM structure and organization and principally in collagen assembly. Secreted phosphoprotein 1 (SPP1), also known as osteopontin-like protein, is a multifunctional secretory acidic glycoprotein (Zhang et al., 2017). Earlier research displayed that POSTN can promote myofibroblast differentiation and type I collagen production, contributing to aberrant lung fibrosis. Serum levels of POSTN may predict the clinical progression of IPF (O'Dwyer and Moore, 2017; Morse et al., 2019; Alimperti and Andreadis, 2015). SPP1 can be secreted by various cells, such as osteoclasts, macrophages, epithelial cells, and endothelial cells. Prior studies reported that low-level local proliferation of macrophages that highly express SPP1 in the normal lung tissue was strikingly increased in IPF lungs, and macrophages that highly express SPP1 importantly contribute to lung fibrosis in IPF34. CDH2, a calcium-dependent cell adhesion protein and also a mesenchymal cell marker, preferentially mediates homotypic cell-cell adhesion by dimerization with a CDH2 chain from another cell (Alimperti and Andreadis, 2015). Previous study showed that CDH2 was involved in epithelial-mesenchymal transition (EMT) which contributes to pulmonary fibrosis (Gao et al., 2019). CTSK displays potent endoprotease activity against fibrinogen (Obermajer et al., 2006), and the expression level of CTSK was up-regulated in the silica induced pulmonary fibrosis model (Brule et al., 2005). These suggested that 11 hub genes identified in the present study play important roles in IPF occurrence.

To explore the prognostic value of these hub genes, survival analysis was performed. It was found that COL1A1, CTSK, MMP1, MMP7, and SPP1 could be potential biomarkers for poor prognosis of IPF patients because the survival rate of IPF patients with high expression levels of COL1A1, CTSK, MMP1, MMP7, and SPP1 was lower than that of those with low expression levels of the abovementioned genes. A number of studies have reported that type I (COL1A1) collagen and matrix metalloproteinases (MMP1 and MMP7) are fibrotic genes associated with the occurrence and prognosis of IPF (Ivan et al., 2008; Giménez et al., 2017; Adegunsoye et al., 2020; Liu et al., 2020; Xu et al., 2020; Mishra et al., 2021). However, further clinical investigation on these genes is warranted.

GO and KEGG pathway enrichment analyses revealed that COL1A1, COL1A2, COL3A1, COL14A1, POSTN, SPP1, MMP1, ASPN, MMP7, CDH2, and CTSK were involved in multiple biological processes related to extracellular matrix organization and collagen metabolism by protein digestion and absorption signaling pathways. In addition, correlation analysis, and PPI as well as cytoHubba plugin analyses showed a highly consistent positive correlation between hub genes in both the training group and the validation group. These results suggest that altered expression of these genes and their crosstalk might impact the development of IPF by modulating the above biological processes and signaling pathways.

The present study analyzed results of seven GEO datasets including 337 IPF samples and 120 control samples to identify possible biomarkers that facilitate elucidation of the underlying molecular mechanism of IPF using a variety of bioinformatics methods (WGCNA, different gene expression analysis, PPI, correlation analysis, etc.). These results were confirmed in 7 GEO datasets, indicating a high reliability and validity. They will shed light on the potential pathogenic mechanism of IPF and guide the development of more potent drugs for IPF.

## Conclusion

In summary, results of the present study suggest that ASPN, CDH2, COL1A1, COL1A2, COL3A1, COL14A1, MMP1, POSTN, SPP1, MMP7, and CTSK are potential biomarkers of IPF. Altered expression of these genes and their cross-talk might impact the development of IPF by modulating IPF-related biological processes and signaling pathways. COL1A1, CTSK, MMP1, MMP7, and SPP1 are positively correlated with IPF prognosis. This study provides further insights into individualized treatment and prognosis for IPF.

## Data Availability

The datasets presented in this study can be found in online repositories. The names of the repository/repositories and accession number(s) can be found in the article/Supplementary Material.

## References

[B1] Adegunsoye A. Alqalyoobi S. Linderholm A. Bowman W. S. Lee C. T. Pugashetti J. V. (2020). Circulating Plasma Biomarkers of Survival in Antifibrotic-Treated Patients with Idiopathic Pulmonary Fibrosis. Chest 158, 1526–1534. 10.1016/j.chest.2020.04.066 32450241 PMC7545483

[B2] Åhrman E. Hallgren O. Malmström L. Hedström U. Malmström A. Bjermer L. (2018). Quantitative Proteomic Characterization of the Lung Extracellular Matrix in Chronic Obstructive Pulmonary Disease and Idiopathic Pulmonary Fibrosis. J. Proteomics 189, 23–33. 10.1016/j.jprot.2018.02.027 29501846

[B3] Alimperti S. Andreadis S. T. (2015). CDH2 and CDH11 Act as Regulators of Stem Cell Fate Decisions. Stem Cel Res. 14, 270–282. 10.1016/j.scr.2015.02.002 PMC4439315 25771201

[B4] Behr J. Nathan S. D. Wuyts W. A. Mogulkoc Bishop N. Bouros D. E. Antoniou K. (2021). Efficacy and Safety of Sildenafil Added to Pirfenidone in Patients with Advanced Idiopathic Pulmonary Fibrosis and Risk of Pulmonary Hypertension: a Double-Blind, Randomised, Placebo-Controlled, Phase 2b Trial. Lancet Respir. Med. 9, 85–95. 10.1016/s2213-2600(20)30356-8 32822614

[B5] Bi S. Chai L. Yuan X. Cao C. Li S. (2017). MicroRNA-98 Inhibits the Cell Proliferation of Human Hypertrophic Scar Fibroblasts via Targeting Col1A1. Biol. Res. 50, 22. 10.1186/s40659-017-0127-6 28629444 PMC5477152

[B6] Brule S. Misson P. Buhling F. Lison D. Huaux F. (2005). Overexpression of Cathepsin K during Silica-Induced Lung Fibrosis and Control by TGF-Beta. Respir. Res. 6, 84. 10.1186/1465-9921-6-84 16045809 PMC1188077

[B7] Chen W. Zhang S. Williams J. Ju B. Shaner B. Easton J. (2020). A Comparison of Methods Accounting for Batch Effects in Differential Expression Analysis of UMI Count Based Single Cell RNA Sequencing. Comput. Struct. Biotechnol. J. 18, 861–873. 10.1016/j.csbj.2020.03.026 32322368 PMC7163294

[B8] Claude J. Harold A. (2018). Idiopathic Pulmonary Fibrosis: Cell Death and Inflammation Revisited. Am. J. Respir. Cel Mol. Biol. 59, 137–138. 10.1165/rcmb.2018-0083ED 29698615

[B9] Cruz-Bermúdez A. Laza-Briviesca R. Vicente-Blanco R. J. García-Grande A. Coronado M. J. Laine-Menéndez S. (2019). Cancer-associated Fibroblasts Modify Lung Cancer Metabolism Involving ROS and TGF-β Signaling. Free Radic. Biol. Med. 130, 163–173. 10.1016/j.freeradbiomed.2018.10.450 30391585

[B11] DePianto D. J. Chandriani S. Abbas A. R. Jia G. N'Diaye E. N. Caplazi P. (2015). Heterogeneous Gene Expression Signatures Correspond to Distinct Lung Pathologies and Biomarkers of Disease Severity in Idiopathic Pulmonary Fibrosis. Thorax 70, 48–56. 10.1136/thoraxjnl-2013-204596 25217476 PMC4472447

[B12] Dong X.-Z. Zhao Z.-R. Hu Y. Lu Y.-P. Liu P. Zhang L. (2020). LncRNA COL1A1-014 Is Involved in the Progression of Gastric Cancer via Regulating CXCL12-CXCR4 axis. Gastric Cancer 23, 260–272. 10.1007/s10120-019-01011-0 31650323

[B13] Gao L. Jiang D. Geng J. Dong R. Dai H. (2019). Hydrogen Inhalation Attenuated Bleomycin‐induced Pulmonary Fibrosis by Inhibiting Transforming Growth Factor‐β1 and Relevant Oxidative Stress and Epithelial‐to‐mesenchymal Transition. Exp. Physiol. 104, 1942–1951. 10.1113/ep088028 31535412

[B14] Ghavami S. Yeganeh B. Zeki A. A. Shojaei S. Kenyon N. J. Ott S. (2018). Autophagy and the Unfolded Protein Response Promote Profibrotic Effects of TGF-Β1 in Human Lung Fibroblasts. Am. J. Physiology-Lung Cell Mol. Physiol. 314, L493–L504. 10.1152/ajplung.00372.2017 PMC5900356 29074489

[B15] Giménez A. Duch P. Puig M. Gabasa M. Xaubet A. Alcaraz J. (2017). Dysregulated Collagen Homeostasis by Matrix Stiffening and TGF-Β1 in Fibroblasts from Idiopathic Pulmonary Fibrosis Patients: Role of FAK/Akt. Ijms 18, 2431. 10.3390/ijms18112431 PMC5713399 29144435

[B16] Hamai K. Iwamoto H. Ishikawa N. Horimasu Y. Masuda T. Miyamoto S. (2016). Comparative Study of Circulating MMP-7, CCL18, KL-6, SP-A, and SP-D as Disease Markers of Idiopathic Pulmonary Fibrosis. Dis. Markers 2016, 4759040. 10.1155/2016/4759040 27293304 PMC4886062

[B17] Huang T.-L. Mu N. Gu J.-T. Shu Z. Zhang K. Zhao J.-K. (2017). DDR2-CYR61-MMP1 Signaling Pathway Promotes Bone Erosion in Rheumatoid Arthritis through Regulating Migration and Invasion of Fibroblast-like Synoviocytes. J. Bone Miner Res. 32, 407–418. 10.1002/jbmr.2993 27653023

[B18] Huang X. Lv D. Yang X. Li M. Zhang H. (2020). m6A RNA Methylation Regulators Could Contribute to the Occurrence of Chronic Obstructive Pulmonary Disease. J. Cel. Mol. Med. 24 (21), 12706–12715. 10.1111/jcmm.15848 PMC7686997 32961012

[B19] Hutchinson J. Fogarty A. Hubbard R. McKeever T. (2015). Global Incidence and Mortality of Idiopathic Pulmonary Fibrosis: a Systematic Review. Eur. Respir. J. 46, 795–806. 10.1183/09031936.00185114 25976683

[B20] Ivan O. Thomas J. Kazuhisa K. (2008). MMP1 and MMP7 as Potential Peripheral Blood Biomarkers in Idiopathic Pulmonary Fibrosis. Plos Med. 5, e93. 10.1371/journal.pmed.0050093 18447576 PMC2346504

[B21] King T. E. Jr. Bradford W. Z. Castro-Bernardini S. Fagan E. A. Glaspole I. Glassberg M. K. (2014). A Phase 3 Trial of Pirfenidone in Patients with Idiopathic Pulmonary Fibrosis. N. Engl. J. Med. 370, 2083–2092. 10.1056/nejmoa1402582 24836312

[B22] Kong Y. Feng Z. C. Zhang Y. L. Liu X. F. Ma Y. Zhao Z. M. (2020). Identification of Immune-Related Genes Contributing to the Development of Glioblastoma Using Weighted Gene Co-expression Network Analysis. Front. Immunol. 11, 1281. 10.3389/fimmu.2020.01281 32765489 PMC7378359

[B23] Li H. Chang H. M. Shi Z. Leung P. C. K. (2020). The P38 Signaling Pathway Mediates the TGF‐β1‐induced Increase in Type I Collagen Deposition in Human Granulosa Cells. FASEB j. 34, 15591–15604. 10.1096/fj.202001377r 32996643

[B24] Liu Y. Wang Y. Lu F. Wang L. Miao L. Wang X. (2020). BTB and CNC Homology 1 Inhibition Ameliorates Fibrosis and Inflammation via Blocking ERK Pathway in Pulmonary Fibrosis. Exp. Lung Res. 26, 1–11. 10.1080/01902148.2020.1849448 33238752

[B25] Liu Y. Zhu M. Geng J. Ban C. Zhang S. Chen W. (2018). Incidence and Radiologic‐pathological Features of Lung Cancer in Idiopathic Pulmonary Fibrosis. Clin. Respir. J. 12, 1700–1705. 10.1111/crj.12732 29094803

[B26] Loitsch S. M. Kippenberger S. Dauletbaev N. Wagner T. O. Bargon J. (1999). Reverse Transcription-Competitive Multiplex PCR Improves Quantification of mRNA in Clinical Samples-Application to the Low Abundance CFTR mRNA. Clin. Chem. 45 (5), 619–624. 10.1093/clinchem/45.5.619 10222347

[B27] Lund J. B. Li S. Baumbach J. Christensen K. Li W. Mohammadnejad A. (2020). Weighted Gene Coregulation Network Analysis of Promoter DNA Methylation on All-Cause Mortality in Old-Aged Birth Cohorts Finds Modules of High-Risk Associated Biomarkers. J. Gerontol. A. Biol. Sci. Med. Sci. 75, 2249–2257. 10.1093/gerona/glaa066 32154558

[B28] Ma H.-P. Chang H.-L. Bamodu O. A. Yadav V. K. Huang T.-Y. Wu A. T. H. (2019). Collagen 1A1 (COL1A1) Is a Reliable Biomarker and Putative Therapeutic Target for Hepatocellular Carcinogenesis and Metastasis. Cancers 11, 786. 10.3390/cancers11060786 PMC6627889 31181620

[B29] Maher T. M. Stowasser S. Nishioka Y. White E. S. Cottin V. Noth I. (2019). Biomarkers of Extracellular Matrix Turnover in Patients with Idiopathic Pulmonary Fibrosis Given Nintedanib (INMARK Study): a Randomised, Placebo-Controlled Study. Lancet Respir. Med. 7, 771–779. 10.1016/S2213-2600(19)30255-3 31326319

[B30] Martinez F. J. Collard H. R. Pardo A. Raghu G. Richeldi L. Selman M. (2017). Idiopathic Pulmonary Fibrosis. Nat. Rev. Dis. Primers 3, 17074. 10.1038/nrdp.2017.74 29052582

[B31] Mishra S. Shah M. I. Udhaya Kumar S. Thirumal Kumar D. Gopalakrishnan C. Al-Subaie A. M. (2021). Network Analysis of Transcriptomics Data for the Prediction and Prioritization of Membrane-Associated Biomarkers for Idiopathic Pulmonary Fibrosis (IPF) by Bioinformatics Approach. Adv. Protein Chem. Struct. Biol. 123, 241–273. 10.1016/bs.apcsb.2020.10.003 33485486

[B32] Morimoto Y. Hirahara K. Kiuchi M. Wada T. Ichikawa T. Kanno T. (2018). Amphiregulin-Producing Pathogenic Memory T Helper 2 Cells Instruct Eosinophils to Secrete Osteopontin and Facilitate Airway Fibrosis. Immunity 49, 134–150. 10.1016/j.immuni.2018.04.023 29958800

[B33] Morse C. Tabib T. Sembrat J. Buschur K. L. Bittar H. T. Valenzi E. (2019). Proliferating SPP1/MERTK-Expressing Macrophages in Idiopathic Pulmonary Fibrosis. Eur. Respir. J. 54, 1802441. 10.1183/13993003.02441-2018 31221805 PMC8025672

[B34] Naik P. K. Bozyk P. D. Bentley J. K. Popova A. P. Birch C. M. Wilke C. A. (2012). Periostin Promotes Fibrosis and Predicts Progression in Patients with Idiopathic Pulmonary Fibrosis. Am. J. Physiology-Lung Cell Mol. Physiol. 303, L1046–L1056. 10.1152/ajplung.00139.2012 PMC3532583 23043074

[B35] Nakajima M. Kizawa H. Saitoh M. Kou I. Miyazono K. Ikegawa S. (2007). Mechanisms for Asporin Function and Regulation in Articular Cartilage. J. Biol. Chem. 282, 32185–32192. 10.1074/jbc.m700522200 17827158

[B10] O'Dwyer D. N. Ashley S. L. Gurczynski S. J. Xia M. Wilke C. Falkowski N. R (2019). Lung Microbiota Contribute to Pulmonary Inflammation and Disease Progression in Pulmonary Fibrosis. Am. J. Respir. Crit. Care Med. 199, 1127–1138. 10.1164/rccm.201809-1650OC 30789747 PMC6515865

[B36] O'Dwyer D. N. Moore B. B. (2017). The Role of Periostin in Lung Fibrosis and Airway Remodeling. Cell Mol Life Sci. 74, 4305–4314. 10.1007/s00018-017-2649-z 28918442 PMC5659879

[B37] Obermajer N. Premzl A. Zavašnik Bergant T. Turk B. Kos J. (2006). Carboxypeptidase Cathepsin X Mediates β2-integrin-dependent Adhesion of Differentiated U-937 Cells. Exp. Cel Res. 312, 2515–2527. 10.1016/j.yexcr.2006.04.019 16774752

[B38] Pei G. Chen L. Zhang W. (2017). WGCNA Application to Proteomic and Metabolomic Data Analysis. Methods Enzymol. 585, 135–158. 10.1016/bs.mie.2016.09.016 28109426

[B39] Raghu G. Remy-Jardin M. Myers J. L. Richeldi L. Ryerson C. J. Lederer D. J. (2018). Diagnosis of Idiopathic Pulmonary Fibrosis. An Official ATS/ERS/JRS/ALAT Clinical Practice Guideline. Am. J. Respir. Crit. Care Med. 198, e44–e68. 10.1164/rccm.201807-1255st 30168753

[B40] Saito S. Alkhatib A. Kolls J. K. Kondoh Y. Lasky J. A. (2019). Pharmacotherapy and Adjunctive Treatment for Idiopathic Pulmonary Fibrosis (IPF). J. Thorac. Dis. 11, S1740–S1754. 10.21037/jtd.2019.04.62 31632751 PMC6783717

[B41] Schäfer S. C. Funke-Chambour M. Berezowska S. (2020). Idiopathische Lungenfibrose - Epidemiologie, Ursachen und klinischer Verlauf. Pathologe 41, 46–51. 10.1007/s00292-019-00747-x 31993696

[B42] Tjin G. White E. S. Faiz A. Sicard D. Tschumperlin D. J. Mahar A. (2017). Lysyl Oxidases Regulate Fibrillar Collagen Remodelling in Idiopathic Pulmonary Fibrosis. Dis. Model. Mech. 10, 1301–1312. 10.1242/dmm.030114 29125826 PMC5719253

[B43] Tran T. Suissa S. (2018). The Effect of Anti-acid Therapy on Survival in Idiopathic Pulmonary Fibrosis: a Methodological Review of Observational Studies. Eur. Respir. J. 51. 10.1183/13993003.00376-2018 29724921

[B44] Tzortzaki E. G. Tischfield J. A. Sahota A. Siafakas N. M. Gordon M. K. Gerecke D. R. (2003). Expression of FACIT Collagens XII and XIV during Bleomycin-Induced Pulmonary Fibrosis in Mice. Anat. Rec. 275A, 1073–1080. 10.1002/ar.a.10120 14613307

[B45] Visse R. Nagase H. (2003). Matrix Metalloproteinases and Tissue Inhibitors of Metalloproteinases. Circ. Res. 92, 827–839. 10.1161/01.res.0000070112.80711.3d 12730128

[B46] Vittal R. Mickler E. A. Fisher A. J. Zhang C. Rothhaar K. Gu H. (2013). Type V Collagen Induced Tolerance Suppresses Collagen Deposition, TGF-Beta and Associated Transcripts in Pulmonary Fibrosis. PLoS One 8, e76451. 10.1371/journal.pone.0076451 24204629 PMC3804565

[B47] Wågsäter D. Zhu C. Björkegren J. Skogsberg J. Eriksson P. (2011). MMP-2 and MMP-9 Are Prominent Matrix Metalloproteinases during Atherosclerosis Development in the Ldlr(-/-)Apob(100/100) Mouse. Int. J. Mol. Med. 28, 247–253. 10.3892/ijmm.2011.693 21567073

[B48] Wan H. Xie T. Xu Q. Hu X. Xing S. Yang H. (2019). Thy-1 Depletion and Integrin β3 Upregulation-Mediated PI3K-Akt-mTOR Pathway Activation Inhibits Lung Fibroblast Autophagy in Lipopolysaccharide-Induced Pulmonary Fibrosis. Lab. Invest. 99 (11), 1636–1649. 10.1038/s41374-019-0281-2 31249375 PMC7102294

[B49] Wang Y. Yella J. Chen J. McCormack F. X. Madala S. K. Jegga A. G. (2017). Unsupervised Gene Expression Analyses Identify IPF-Severity Correlated Signatures, Associated Genes and Biomarkers. BMC Pulm. Med. 17, 133. 10.1186/s12890-017-0472-9 29058630 PMC5649521

[B50] Wei C. An-Lin C. Marco B. Chou W. C. Cheng A. L. Brotto M. (2014). Visual Gene-Network Analysis Reveals the Cancer Gene Co-expression in Human Endometrial Cancer. BMC Genomics 15, 300. 10.1186/1471-2164-15-300 24758163 PMC4234489

[B51] Xu Y. Mizuno T. Sridharan A. Du Y. Guo M. Tang J. (2016). Single-cell RNA Sequencing Identifies Diverse Roles of Epithelial Cells in Idiopathic Pulmonary Fibrosis. JCI Insight 1, e90558. 10.1172/jci.insight.90558 27942595 PMC5135277

[B52] Xu Z. Mo L. Feng X. Huang M. Li L. (2020). Using Bioinformatics Approach Identifies Key Genes and Pathways in Idiopathic Pulmonary Fibrosis. Medicine (Baltimore) 99 (36), e22099. 10.1097/md.0000000000022099 32899090 PMC7478566

[B53] Yu D. H. Ruan X. L. Huang J. Y. Liu X. P. Ma H. L. Chen C. (2020). Analysis of the Interaction Network of Hub miRNAs-Hub Genes, Being Involved in Idiopathic Pulmonary Fibers and its Emerging Role in Non-small Cell Lung Cancer. Front. Genet. 11, 302. 10.3389/fgene.2020.00302 32300359 PMC7142269

[B54] Zhang X. Liu H. Hock T. Thannickal V. Sanders Y. (2013). Histone Deacetylase Inhibition Downregulates Collagen 3A1 in Fibrotic Lung Fibroblasts. Ijms 14, 19605–19617. 10.3390/ijms141019605 24084714 PMC3821575

[B55] Zhang Y. Du W. Chen Z. Xiang C. (2017). Upregulation of PD-L1 by SPP1 Mediates Macrophage Polarization and Facilitates Immune Escape in Lung Adenocarcinoma. Exp. Cel Res. 359, 449–457. 10.1016/j.yexcr.2017.08.028 28830685

