# Supplementary figures and images for: Identification of Hub Genes and Pathways Associated With Idiopathic Pulmonary Fibrosis via Bioinformatics Analysis
Source: Front Mol Biosci. 2021 Aug 12;8:711239. doi: 10.3389/fmolb.2021.711239 (PMC8406749; doi:10.3389/fmolb.2021.711239)

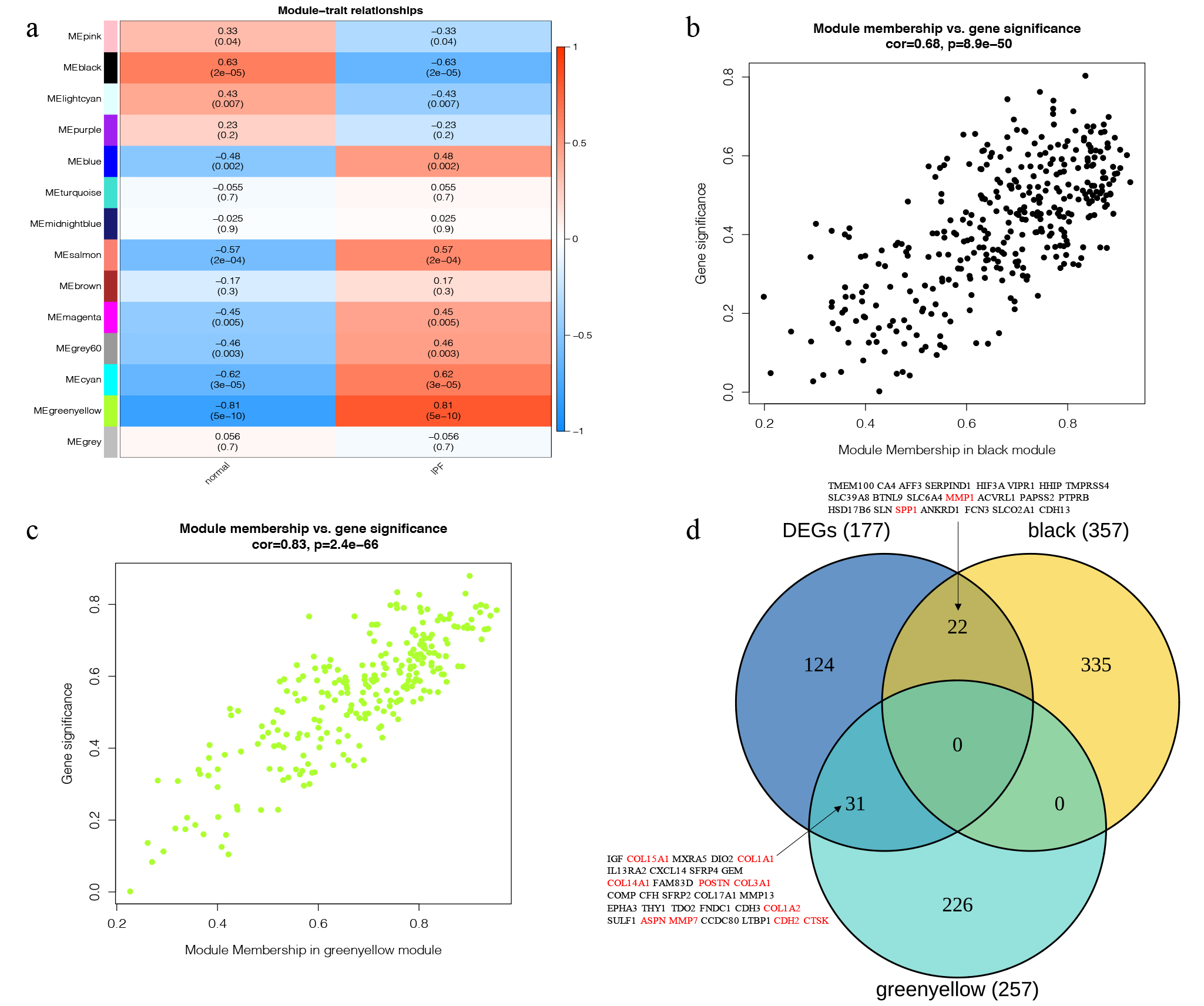

Supplement: Supplementary file 3 [file Image1.JPEG]
