# Supplementary material for: Identification of Hub Genes and Pathways Associated With Idiopathic Pulmonary Fibrosis via Bioinformatics Analysis
Source: Front Mol Biosci. 2021 Aug 12;8:711239. doi: 10.3389/fmolb.2021.711239 (PMC8406749; doi:10.3389/fmolb.2021.711239)
Supplement: Supplementary file 8 [file DataSheet1.docx]

**Supplementary Figure S1 validation of hub genes in GSE10667.** (**a**) The module-trait relationships were evaluated by correlating module eigengenes with clinical traits. (**b**) Scatter plots of module eigengenes in the black modules. (**c**) Scatter plots of module eigengenes in the greenyellow modules. (**d**) The Venn diagram demonstrating twelve hub genes.

**Supplementary Table 1** KEGG pathway analysis of key genes in IPF

| ID | Term | Count | *p*.adjust | Genes |
| --- | --- | --- | --- | --- |
| hsa04974 | protein digestion and absorption | 7 | 0.000127 | COL14A1, COL15A1, COL17A1, COL1A1, COL1A2, COL3A1, CPA3 |
| hsa04512 | ECM-receptor interaction | 4 | 0.069808 | COL1A1, COL1A2, COMP, SPP1 |
| hsa04933 | AGE-RAGE signaling pathway in diabetic complications | 4 | 0.074638 | COL1A1, COL1A2, COL3A1, VCAM1 |
| hsa00450 | selenocompound metabolism | 2 | 0.080115 | INMT, PAPSS2 |
| hsa04611 | platelet activation | 4 | 0.080115 | COL1A1, COL1A2, COL3A1, P2RY1 |
| hsa04510 | focal adhesion | 5 | 0.080115 | COL1A1, COL1A2, COMP, IGF1, SPP1 |
| hsa04926 | relaxin signaling pathway | 4 | 0.080115 | COL1A1, COL1A2, COL3A1, MMP1 |
| hsa04514 | cell adhesion molecules | 4 | 0.116072 | CDH2, CDH3, CLDN1, VCAM1 |
| hsa04350 | TGF-beta signaling pathway | 3 | 0.173514 | ID1, LTBP1, SMAD6 |
| hsa05146 | amoebiasis | 3 | 0.194115 | COL1A1, COL1A2, COL3A1 |

**Supplementary Table 2** GO function analyses for proteins interacting with COL1A1, COL1A2, COL3A1, Col14A1, COL15A1, POSTN, SPP1, MMP1, ASPN, CDH2, MMP7, and CTSK.

| Category | Term | Description | Count | Adj_pval |
| --- | --- | --- | --- | --- |
| **COL1A1** | | | | |
| Biological process | GO:0030198 | extracellular matrix organization | 17/72 | 5.25E-11 |
| Biological process | GO:0043062 | extracellular structure organization | 17/72 | 2.43E-10 |
| Biological process | GO:0071559 | response to TGF beta | 12/72 | 1.28E-07 |
| Biological process | GO:0031589 | cell-substrate adhesion | 13/72 | 2.67E-07 |
| Biological process | GO:0071560 | cellular response to EGF beta stimulus | 11/72 | 7.56E-07 |
| Biological process | GO:0032963 | collagen metabolic process | 8/72 | 3.36E-06 |
| Biological process | GO:0001503 | ossification | 12/72 | 7.24E-06 |
| Biological process | GO:0030199 | collagen fibril organization | 6/72 | 1.14E-05 |
| Molecular function | GO:0005201 | extracellular matrix structural constituent | 13/72 | 1.49E-11 |
| Molecular function | GO:0030020 | extracellular matrix structural constituent conferring tensile strength | 6/72 | 9.64E-07 |
| Molecular function | GO:0048407 | platelet-derived growth factor binding | 3/72 | 0.000445 |
| Molecular function | GO:0019838 | growth factor binding | 6/72 | 0.000654 |
| **COL1A2** | | | | |
| Biological process | GO:0030198 | extracellular matrix organization | 17/72 | 5.25E-11 |
| Biological process | GO:0043062 | extracellular structure organization | 17/72 | 2.43E-10 |
| Biological process | GO:0071559 | response to TGF beta | 12/72 | 1.28E-07 |
| Biological process | GO:0007178 | transmembrane receptor protein | 13/72 | 2.67E-07 |
| Biological process | GO:0071560 | cellular response to EGF beta stimulus | 11/72 | 7.56E-07 |
| Biological process | GO:0032963 | collagen metabolic process | 8/72 | 3.36E-06 |
| Biological process | GO:0001503 | ossification | 12/72 | 7.24E-06 |
| Biological process | GO:0030199 | collagen fibril organization | 6/72 | 1.14E-05 |
| Molecular function | GO:0005201 | extracellular matrix structural constituent | 13/72 | 1.49E-11 |
| Molecular function | GO:0030020 | extracellular matrix structural constituent conferring tensile strength | 6/72 | 9.64E-07 |
| Molecular function | GO:0048407 | platelet-derived growth factor binding | 3/72 | 0.000445 |
| Molecular function | GO:0019838 | growth factor binding | 6/72 | 0.000654 |
| **COL3A1** | | | | |
| Biological process | GO:0030198 | extracellular matrix organization | 17/72 | 5.25E-11 |
| Biological process | GO:0043062 | extracellular structure organization | 17/72 | 2.43E-10 |
| Biological process | GO:0071559 | response to TGF beta | 12/72 | 1.28E-07 |
| Biological process | GO:0007178 | transmembrane receptor protein | 13/72 | 2.67E-07 |
| Biological process | GO:0031589 | cell-substrate adhesion | 13/72 | 2.67E-07 |
| Biological process | GO:0071560 | cellular response to EGF beta stimulus | 11/72 | 7.56E-07 |
| Biological process | GO:0030199 | collagen fibril organization | 6/72 | 1.14E-05 |
| Molecular function | GO:0005201 | extracellular matrix structural constituent | 13/72 | 1.49E-11 |
| Molecular function | GO:0050839 | cell adhesion molecule binding | 15/72 | 9.29E-08 |
| Molecular function | GO:0030020 | extracellular matrix structural constituent conferring tensile strength | 6/72 | 9.64E-07 |
| Molecular function | GO:0005178 | integrin binding | 8/72 | 2.89E-06 |
| Molecular function | GO:0048407 | platelet-derived growth factor binding | 3/72 | 0.000445 |
| Molecular function | GO:0019838 | growth factor binding | 6/72 | 0.000654 |
| **COL14A1** | | | | |
| Biological process | GO:0030198 | extracellular matrix organization | 17/72 | 5.25E-11 |
| Biological process | GO:0043062 | extracellular structure organization | 17/72 | 2.43E-10 |
| Biological process | GO:0030199 | collagen fibril organization | 6/72 | 1.14E-05 |
| Molecular function | GO:0005201 | extracellular matrix structural constituent | 13/72 | 1.49E-11 |
| Molecular function | GO:0030020 | extracellular matrix structural constituent conferring tensile strength | 6/72 | 9.64E-07 |
| **COL15A1** | | | | |
| Biological process | GO:0030198 | extracellular matrix organization | 17/72 | 5.25E-11 |
| Biological process | GO:0043062 | extracellular structure organization | 17/72 | 2.43E-10 |
| Biological process | GO:0030199 | collagen fibril organization | 6/72 | 1.14E-05 |
| Biological process | GO:0032963 | collagen metabolic process | 8/72 | 3.36E-06 |
| Molecular function | GO:0005201 | extracellular matrix structural constituent | 13/72 | 1.49E-11 |
| Molecular function | GO:0030020 | extracellular matrix structural constituent conferring tensile strength | 6/72 | 9.64E-07 |
| **POSTN** | | | | |
| Biological process | GO:0030198 | extracellular matrix organization | 17/72 | 5.25E-11 |
| Biological process | GO:0043062 | extracellular structure organization | 17/72 | 2.43E-10 |
| Biological process | GO:0071559 | response to TGF beta | 12/72 | 1.28E-07 |
| Biological process | GO:0031589 | cell-substrate adhesion | 13/72 | 2.67E-07 |
| Biological process | GO:0071560 | cellular response to EGF beta stimulus | 11/72 | 7.56E-07 |
| Molecular function | GO:0005201 | extracellular matrix structural constituent | 13/72 | 1.49E-11 |
| Molecular function | GO:0050839 | cell adhesion molecule binding | 15/72 | 9.29E-08 |
| Molecular function | GO:0005539 | glycosaminoglycan binding | 7/72 | 0.001107 |
| Molecular function | GO:0008201 | heparin binding | 6/72 | 0.001599 |
| **SPP1** | | | | |
| Biological process | GO:0030198 | extracellular matrix organization | 17/72 | 5.25E-11 |
| Biological process | GO:0043062 | extracellular structure organization | 17/72 | 2.43E-10 |
| Biological process | GO:0001503 | ossification | 12/72 | 7.24E-06 |
| Molecular function | GO:0050839 | cell adhesion molecule binding | 15/72 | 9.29E-08 |
| Molecular function | GO:0005178 | integrin binding | 8/72 | 2.89E-06 |
| **MMP1** | | | | |
| Biological process | GO:0030198 | extracellular matrix organization | 17/72 | 5.25E-11 |
| Biological process | GO:0043062 | extracellular structure organization | 17/72 | 2.43E-10 |
| Biological process | GO:0032963 | collagen metabolic process | 8/72 | 3.36E-06 |
| **ASPN** | | | | |
| Biological process | GO:0071559 | response to TGF beta | 12/72 | 1.28E-07 |
| Biological process | GO:0007178 | transmembrane receptor protein | 13/72 | 2.67E-07 |
| Biological process | GO:0071560 | cellular response to EGF beta stimulus | 11/72 | 7.56E-07 |
| Biological process | GO:0001503 | ossification | 12/72 | 7.24E-06 |
| Molecular function | GO:0005201 | extracellular matrix structural constituent | 13/72 | 1.49E-11 |
| **CDH2** | | | | |
| Molecular function | GO:0050839 | cell adhesion molecule binding | 15/72 | 9.29E-08 |
| **MMP7** | | | | |
| Biological process | GO:0030198 | extracellular matrix organization | 17/72 | 5.25E-11 |
| Biological process | GO:0043062 | extracellular structure organization | 17/72 | 2.43E-10 |
| Biological process | GO:0032963 | collagen metabolic process | 8/72 | 3.36E-06 |
| Molecular function | GO:0005539 | glycosaminoglycan binding | 7/72 | 0.001107 |
| Molecular function | GO:0008201 | heparin binding | 6/72 | 0.001599 |
| **CTSK** | | | | |
| Biological process | GO:0030198 | extracellular matrix organization | 17/72 | 5.25E-11 |
| Biological process | GO:0043062 | extracellular structure organization | 17/72 | 2.43E-10 |
| Biological process | GO:0032963 | collagen metabolic process | 8/72 | 3.36E-06 |
| Biological process | GO:0001503 | ossification | 12/72 | 7.24E-06 |

**Supplementary Table 3** The mRNA expression levels of 12 key genes in a variety of samples of idiopathic pulmonary fibrosis.

| Genes | Function (<https://www.genecards.org/>) | Group | Sample type | The mRNA expression level | Ref |
| --- | --- | --- | --- | --- | --- |
| COL1A1 | Fibrillar forming collagen. | IPF vs. control | Lung tissue | Up | ^17^ |
| COL1A2 | Fibril forming, down-regulated c-Myc target gene. | TGF-β1-exposure mice vs. PBS-exposure mice | Airway fibroblasts and lung fibroblasts | Up | ^39^ |
| COL3A1 | The major ligand of ADGRG1, activaes the RhoA pathway. | IPF vs. control | Lung tissue | Up | ^40^ |
| COL14A1 | Integrate collagen bundles. | Bleomycin-exposure mice vs. saline-exposure mice | Lung tissue | Up | ^41^ |
| COL15A1 | Inhibit angiogenesis. | Unknown | Unknown | Unknown |  |
| POSTN | Induces cell attachment, spreading and adhesion. | IPF vs. control | Lung fibroblasts | Up | ^42^ |
| SPP1 | Acts as a cytokine involved in enhancing production of interferon-gamma | House dust mite -exposure mice vs. PBS-exposure mice | Lung tissue | Up | ^43^ |
| MMP1 | May be involved in tissue injury and remodeling | IPF vs. control | Blood; bronchoalveolar lavage | Up | ^44^ |
| ASPN | Inhibits BMP2 binding to BMPR1B/BMP type-1B receptor. | IPF vs. control | Lung tissue | Up | ^29^ |
| CDH2 | Mediates homotypic cell-cell adhesion. | Bleomycin-exposure mice vs. saline-exposure mice | Lung tissure | Up | ^45^ |
| MMP7 | Degrade casein, gelatins of types I, III, IV and V, and fibronectin. | IPF vs. control | Blood | Up | ^36^ |
| CTSK | Endoprotease activity against fibrinogen. | Silica-exposure mice vs. PBS-exposure mice | Lung tissue | Up | ^46^ |

Note: IPF, idiopathic pulmonary fibrosis.
